# Silver Nanoparticles: A Comprehensive Review of Synthesis Methods and Chemical and Physical Properties

**DOI:** 10.3390/nano14181527

**Published:** 2024-09-20

**Authors:** Hatice Duman, Furkan Eker, Emir Akdaşçi, Anna Maria Witkowska, Mikhael Bechelany, Sercan Karav

**Affiliations:** 1 Department of Molecular Biology and Genetics, Çanakkale Onsekiz Mart University, Çanakkale 17100, Türkiye; hatice.duman@comu.edu.tr (H.D.); furkan.eker@stu.comu.edu.tr (F.E.); emirakdasci@gmail.com (E.A.); 2 Department of Food Biotechnology, Medical University of Bialystok, 15-089 Bialystok, Poland; anna.witkowska@umb.edu.pl; 3 Institut Européen des Membranes (IEM), UMR 5635, University of Montpellier, ENSCM, CNRS, F-34095 Montpellier, France; 4 Functional Materials Group, Gulf University for Science and Technology (GUST), Masjid Al Aqsa Street, Mubarak Al-Abdullah 32093, Kuwait

**Keywords:** silver nanoparticles, antimicrobial effects, optical properties, chemical synthesis, physical synthesis, bio-based synthesis, toxicity mechanisms

## Abstract

Recently, silver nanoparticles (NPs) have attracted significant attention for being highly desirable nanomaterials in scientific studies as a result of their extraordinary characteristics. They are widely known as effective antibacterial agents that are capable of targeting a wide range of pathogens. Their distinct optical characteristics, such as their localized surface plasmon resonance, enlarge their utilization, particularly in the fields of biosensing and imaging. Also, the capacity to control their surface charge and modify them using biocompatible substances offers improved durability and specific interactions with biological systems. Due to their exceptional stability and minimal chemical reactivity, silver NPs are highly suitable for a diverse array of biological applications. These NPs are produced through chemical, biological, and physical processes, each of which has distinct advantages and disadvantages. Chemical and physical techniques often encounter issues with complicated purification, reactive substances, and excessive energy usage. However, eco-friendly biological approaches exist, even though they require longer processing times. A key factor affecting the stability, size distribution, and purity of the NPs is the synthesis process selected. This review focuses on how essential it is to choose the appropriate synthesis method in order to optimize the characteristics and use of silver NPs.

## 1. Introduction

Nanoparticles (NPs) are small materials that range from 1 to 100 nm in size. They have unique physical and chemical properties, along with biological aspects, and their application has been extended to various fields. Their small size and large surface-area-to-volume ratio comprise their unique characteristics, so they greatly differ from their bulkier counterparts [1].

There are various types of NPs that are currently being studied in a large area of application. Among these, silver NPs have gained attention due to their efficient activities, especially their antimicrobial activity. Their antimicrobial characteristics enable them to possess bactericidal, fungicidal, and virucidal properties [2]. Even though it is not fully demonstrated yet, the primary mechanisms behind the antimicrobial activity of silver NPs involve the release of silver ions that can interact with microbial membranes, disrupting cellular function and leading to the generation of reactive oxygen species (ROS). These multiple proposed mechanisms make silver NPs efficient against various pathogens, including the ones that are resistant to antibiotics and multiple drugs [3]. Similarly, these mechanisms are emphasized in various types of applications as well. For instance, antimicrobial activity is crucial in agricultural, wound healing, and dental applications [4,5,6]. Additionally, the release of silver ions, membrane penetration, and intracellular activity of silver NPs are significant in anticancer and bioimaging applications [7].

On the other hand, the synthesis of silver NPs strongly influences their wide-ranging applications. In other words, these factors are crucial in controlling silver NP applications. Since silver NPs exhibit significant electrical and thermal conductivity, they have been utilized in electronics, where they are used in conductive inks, adhesives, and electronic components [8]. They are also utilized for thermal management purposes and included in energy storage devices, thanks to their efficient heat conduction [9]. Furthermore, silver NPs possess notable optical properties, including their conductivity, because of the localized surface plasmon resonance (LSPR) phenomenon. This effect occurs when conduction electrons on the NP surface resonate with incident light and convey strong absorbance and scattering capabilities to silver NPs. Based on this, they are highlighted as potential materials to be utilized in biosensing, bioimaging, and photothermal therapies [10]. They can also enhance the sensitivity of surface-enhanced Raman scattering (SERS) with their LSPR characteristics. Their application to SERS enables the detection of low analyte concentrations that are useful in environmental monitoring and medical diagnostics [11]. Silver NPs can also be functionalized with various molecules, which allows the surfaces to be modified for the desired characteristics. This modification can enhance the stability, biocompatibility, and target specificity of the NPs, thus improving their application in the biomedical area [12].

The physicochemical characteristics of silver NPs have attracted great attention, especially in biological uses. Various techniques, such as chemical, biological, and physical processes, are used in the synthesis of silver NPs [13]. Depending on the chosen synthesis method, various properties of NPs are directly influenced, such as stability, purity, and size distribution. Despite their effectiveness, hazardous reactants can be used in traditional chemical and physical methods. In addition, they have difficult purification procedures, low conversion efficiencies, high energy and cost consumption, and can pose environmental hazards. Conversely, bio-based synthesis techniques (involving microorganisms, plants, or algae) provide an environmentally friendly approach to NP synthesis. These organisms function as effective reducing and stabilizing agents, therefore obviating the necessity for hazardous chemicals. Furthermore, silver NPs produced by biological synthesis demonstrate excellent colloidal stability because of inherent capping agents, such as proteins and polysaccharides. These agents suppress agglomeration and improve the dispersion of silver NPs in water-based solutions. Together with features of the synthesis procedure, they can exhibit improved or combined antibacterial characteristics as a result of the existence of naturally occurring bioactive compounds on their surfaces. Although biological methods are economical since they utilize renewable resources such as plant extracts or microbial cultures, scaling them up can be difficult because of material variability and batch-to-batch uniformity. Still, they also possess certain drawbacks, such as the requirement for careful culture maintenance and lengthier processing periods [14]. Top-down and bottom-up methodologies are typically used to synthesize NPs. Bottom-up synthesis methods, such as chemical vapor deposition and bio-based synthesis, use atomic and molecular assembly to generate NPs. This offers a promising approach to non-toxic and economical synthesis. To have the desired characteristics in the synthesized NPs, it is crucial to choose the process that best balances the advantages and constraints [15,16].

**Figure 1:**
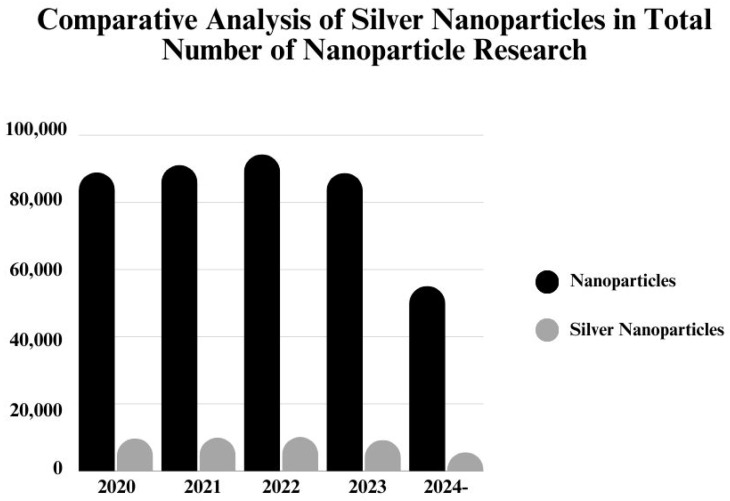
Comparison of silver NP-related documents with respect to the total number of NP research [17].

Currently, NPs have emerged as one of most studied materials in scientific research. Based on the data provided by the Web of Science Core Collection, more than 500.000 published documents, including both patents and papers, were reported in the past 5 years (Figure 1) [17]. Specifically, 54.070 of these documents are related to silver NPs, which comprise 10% of the total published documents in the field of nanomaterials. In recent publications related to silver NPs, researchers evaluated various synthesis methods, antimicrobial properties, development of drug delivery systems, and toxicological effects. One of the primary focuses was the green-synthesized approaches, with the aim of mitigating the adverse effects associated with silver NPs. Hence, considering these latest studies and remarkable statistics, our aim is to contribute to NP research with a comprehensive review by addressing the recent advances with silver NPs.

**Figure 2:**
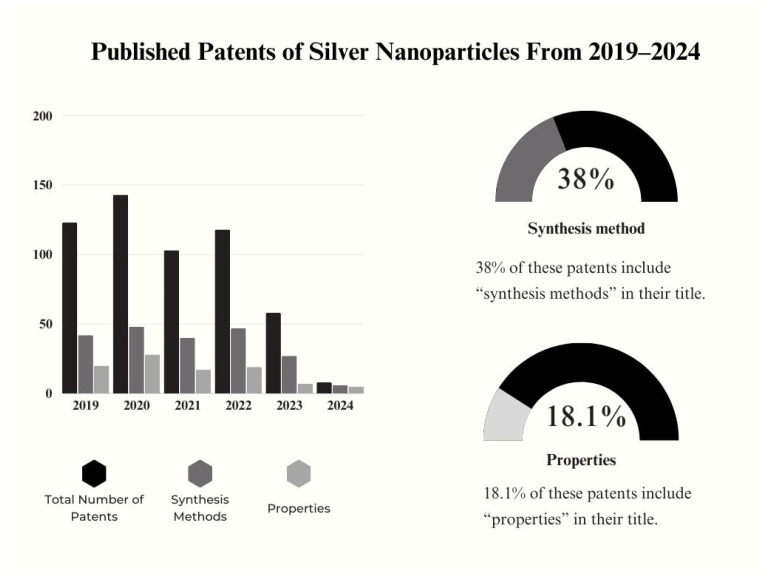
Graph representing the number of patents containing keywords that are related to physical and chemical properties and “synthesis methods” in comparison to the total number of patents published from 2019 to 2024 [18].

Additionally, we have analyzed the number of patents published in the last five years and their relation to different areas (Figure 2) [18]. Considering more than 500 documents, statistics have showed that those including “synthesis methods” and “properties” (associated with both physical and chemical aspects) in their title have comprised 38 and 18.1 percent of the total number of registered patents. Considering the recent advances and predominant utilization of green synthesis methods, the higher ratio of the registered method-based patents might indicate the extensive potential of an eco-friendly industrial commercialization in the near future. Excluding 2024, the number of total documents published in the last five years maintains a high level of research. Despite the reduction in the number of patent registrations in the last year, the total number of research papers each year is still high. Since the importance of green synthesis increases each year, we might assume that silver NP research has shifted to innovative approaches and led to a reduction in the patent registrations.

The current developments in synthesis methods, particularly green-based approaches, and the properties of silver NPs, along with recent examples of their applications, have been analyzed. Patents from the last five years, particularly those associated with environmentally friendly synthesis methods, were evaluated, highlighting the increasing prevalence of methods and property-based patents. Given the high number of recent publications, this review provides insights into silver NPs, focusing on the crucial characteristics that will significantly impact their future applications. For example, recent developments in silver NP-based bioimaging and biosensing research are increasingly focused on creating more sensitive, specific, and innovative systems. In particular, many recent studies show promising potential for silver NP applications in cancer research. Considering the variables that affect the optical properties and effectiveness of silver NPs, a review of recent perspectives and findings on their properties and synthesis methods is essential to guide future research in this area. For the advancement and commercialization of silver NP-based sensors, up-to-date perspectives and findings are critical elements that need to be present in the current literature.

The same situation applies to the antibacterial applications of silver NPs, as their antimicrobial properties form the backbone of most silver NP-based applications. There is a shift in the use of silver NPs for antimicrobial, wound healing, and drug delivery purposes toward incorporating them into hybrid materials, such as nanocomposites or complexes, particularly with polymers. These hybrid materials are preferred not only for their enhanced efficiency but also for their ability to reduce potential cytotoxicity. This review has covered the factors affecting these applications on a large scale, based on current knowledge, and has also highlighted a future roadmap for this field, emphasizing the importance of the characteristics and production methods of silver NPs.

In conclusion, the multifaceted characteristics of silver NPs and their importance in several fields are discussed, encompassing their antimicrobial effects and improved optical qualities in this article. The text offers a thorough analysis of the mechanisms by which silver NPs contribute to the progress of nanotechnology. Together with the detailed physical and chemical properties of silver NPs and their recent application examples, the main synthesizing approaches are also investigated. The article extensively examines the application areas, benefits, and drawbacks of NP synthesis techniques, as categorized into three primary categories. Exemplary examples are included to facilitate comprehensive examination. This paper’s primary and unique characteristic is its comprehensive examination of patent trends pertaining to silver NPs, with a specific emphasis on synthesis techniques and material characteristics. A comprehensive analysis of silver NP research and prospective uses is presented in this article, with a specific emphasis on synthesis and patent trends. The text emphasizes the increasing capacity for environmentally friendly industrial applications and proposes notable advancements in this area, suggesting a rising interest in green approaches for commercialization.

## 2. Properties of Silver Nanoparticles

**Figure 3:**
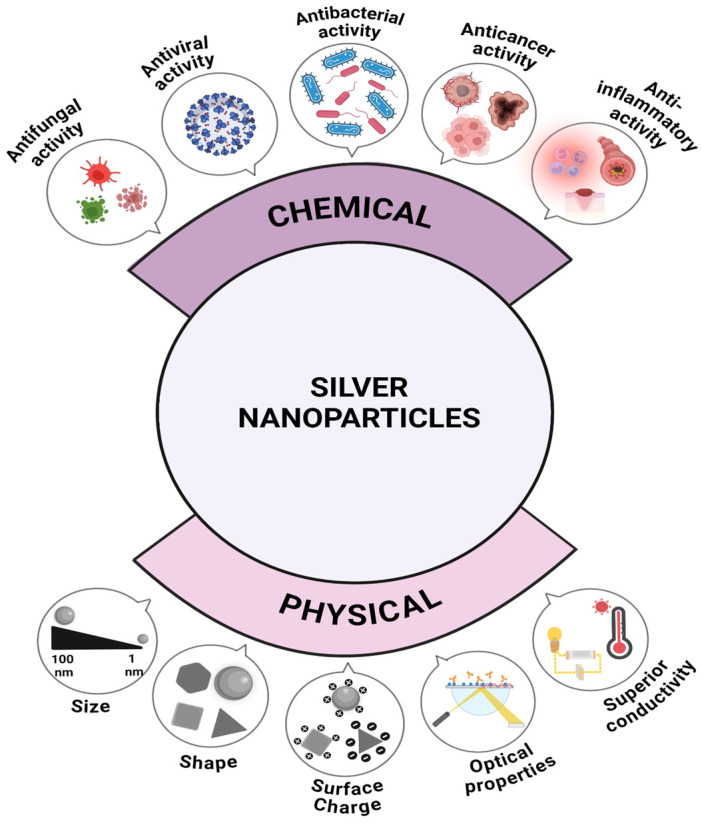
Illustration of physical and chemical traits of silver NPs [23].

Reflecting typical NP traits, silver NPs exhibit distinct physical and chemical properties due to their small size and high surface-area-to-volume ratio (Figure 3) [13]. These properties include high electrical and thermal conductivity, antibacterial activity, and optical characteristics, which mainly depend on their shape, size, surface chemistry, composition, coating, and agglomeration [19].

In line with that, the synthesis method is also essential in defining the characteristics of silver NPs. For instance, both the advantages and disadvantages of silver NP synthesis methods through physical, chemical, and biological approaches were demonstrated in a recent review [20]. Moreover, compared to other types of metals, silver NPs exhibit stable and chemically less reactive characteristics. These properties make them remarkable agents for many biological applications, including antibacterial coatings, biosensors, bioimaging, wound healing, diabetes treatment, and cancer therapy [21]. Properties of silver NPs are also related to their toxicity potential, which has been extensively discussed in the current literature [22]. Nevertheless, detailed examples of these negative effects, including their mechanisms and potential health risks, will be discussed in the toxicity section.

In this section, we have explored the diverse properties of silver NPs and their significance in various disciplines. From their exceptional antibacterial activities to enhanced optical traits, silver NPs have been included in much innovative research and many applications. Regarding the current research background, this section aims to provide a comprehensive understanding of how silver NPs contribute to advances in nanotechnology with their unique characteristics.

### 2.1. Size

Size is one of the important factors that determines silver NPs physical, chemical, and biological properties [24]. It significantly influences their surface-area-to-volume ratio, optical characteristics, and conductivity, as well as creating opportunities in various fields [25]. Their small size improves reactivity and interaction with the surrounding molecules, in solution or on surfaces [26]. This increased reactivity is mainly advantageous in catalysis and sensing studies, where silver NPs are employed as catalysts due to their size-dependent surface properties [27].

In addition, their small size allows them to penetrate biological systems more effectively and enables their utilization in targeted drug delivery and imaging in biomedical applications [28]. Still, their high-penetration efficiency can also result in increased toxicity, indicating a correlation between the size and the toxicity of silver NPs. Also, Cho et al. revealed that smaller silver NPs (with diameters around 10 nm) exhibited stronger hepatotoxic effects in mice models compared to larger counterparts (60 nm and 100 nm) [29]. Considering these, it becomes evident that the size of NPs greatly affect their physical attributes, biological effects, and toxicity. Hence, a careful consideration of size in the synthesis process must be taken into account.

### 2.2. Shape

Silver NPs can be synthesized in various shapes, including spherical, rod-shaped, triangular, cubic, wire-like, and star-shaped [30]. Many properties, such as optical, catalytic, and electrical, are influenced by the shape of the silver NPs [25]. For instance, the shape and size of the silver NPs significantly strengthen their interactions with biological systems. Thus, they are suitable for employment in the biomedical area, especially in drug delivery systems and coatings [31,32]. Additionally, the antimicrobial activity may be influenced by the rate of silver ions released from silver NPs. In this manner, spherical NPs are highly favored since they are capable of releasing silver ions more efficiently due to their higher surface-area-to-volume ratio in comparison to other shapes, such as triangular plates and disks. This increased ion release enhances silver NPs antimicrobial activity by allowing a more effective disruption of bacterial cell membranes and interference with cellular processes [33]. Therefore, spherical silver NPs are generally preferred for biomedical contexts because of their uniformity, facile synthesis, and remarkable antibacterial properties. However, triangular-shaped silver NPs have also gained attention for their distinct antibacterial properties, which have been extensively studied and compared to their spherical counterparts [34].

Furthermore, silver NPs of various shapes, such as nanospheres, nanocubes, and nanoprisms, were investigated in terms of their electrochemical behavior in alkaline solutions. Findings showed that nanoprisms enhanced catalytic activity compared to nanospheres and nanocubes. The results highlight the crucial role of shape-dependent electrochemical properties, especially in applications such as sensing and fuel cells [35]. Moreover, another study compared the skin permeability of various shapes of silver NPs, including spherical, rod-shaped, and triangular. It was revealed that rod-shaped silver NPs have the highest permeability compared to other types. These findings were also demonstrated in *in vivo* mice experiments, as rod-shaped silver NPs accumulated the most in the blood after topical application [36].

To summarize, since the properties of silver NPs are influenced by their shape, these shapes offer unique advantages, such as enhanced antibacterial efficiency, catalytic activity, penetration capability, and optical properties [37,38,39]. Therefore, determining the shape during the synthesis process can enhance the utilization of silver NPs in numerous applications.

### 2.3. Surface Charge

The surface charge is one of the important properties of NPs that significantly affects their stability and interactions with other molecules. Manipulating the surface charge allows for precise control of NP behavior in different environments, thus influencing their aggregation, solubility, and reactivity. In line with that, the surface charge is also considered essential in biomedical applications, where modified surface charges improve targeting, uptake, and therapeutic efficiency [40]. In addition, surface charges can also be manipulated through pH adjustments as well as surface functionalization. Overall, these charges influence their dispersion in solvents or matrices and also their interactions with other biomolecules or surfaces [41].

For example, positively charged silver NPs that have achieved thorough functionalization with polymers (polyethyleneimine or chitosan) have been shown to enhance silver NPs’ dispersion in aqueous solutions through electrostatic repulsion [42,43]. Moreover, modifying surface charges influences the biological activities of silver NPs. As an example, Abbas et al. investigated how different surface charges on silver NPs affect their ability to combat bacteria such as *Staphylococcus aureus* (*S. aureus*) and *Escherichia coli* (*E. coli*). Findings indicated positively charged silver NPs were the most bactericidal, creating larger zones of inhibition and requiring lower concentrations to be effective compared to their neutral and negatively charged counterparts [44]. Similarly, El Badawy et al. studied the effect of surface charges concerning the toxicity of silver NPs toward the *Bacillus* species. Focusing on silver NPs with varying surface charges (positive, negative, and neutral), they indicated a relationship between surface charges and toxicity. In particular, positively charged silver NPs were the most toxic among their counterparts, as they significantly influenced bacterial oxygen consumption and cell viability [45]. Briefly, modulating surface charges remarkably influences the stability, dispersion, and reactivity of silver NPs. Thus, it might be considered an effective approach to optimize their utilization in biomedical applications.

### 2.4. Electrical Conductivity and Melting Point

Bulk silver has some of the highest electrical conductivity at room temperature compared to other metals due to the atomic structure and existence of free electrons in silver [46]. Similar to other metals, silver has a single valence electron in the outermost shell that is not tightly bound to the nucleus [47]. Therefore, it allows the electrons to move freely throughout the metals, allowing a conductive pathway. In other words, when an electric field is applied, these electrons will move easily, which allows the electric current to flow with minimal resistance. These free movements of the electrons are the primary factor of silver’s electrical conductivity and its utilization in conductive composite formulations with both organic and inorganic materials [48]. The same factors are also applied to silver NPs, where the free electrons are involved in their electrical conductivity [49]. Moreover, the electrical conductivity of silver NPs is highly influenced by their zeta potential, an essential parameter characterizing the electrical potential difference across the NPs surface relative to the surrounding medium [50]. Zeta potential serves as a key indicator of stability, since it indicates a high electrostatic repulsion between particles that prevents agglomeration in higher values (both at negative and positive values) [51].

Silver NPs exhibit reduced melting points due to their smaller size, which is considered advantageous compared to their bulkier counterparts [52]. More precisely, reducing the size of the particles causes an increase in the ratio of surface area to volume, which in turn enhances the amount of free energy on the particle’s surface [53]. This property enables lower temperature methods and improves practicality for applications such as nanocomposite materials for advanced electrical devices and sensors, which require significant melting temperatures [54]. The somewhat lower melting point of silver NPs holds significant potential for conducting electrical research due to its ability to enhance the sintering process, which involves the fusion of particles to create cohesive structures [55]. This process enhances electrical performance by creating continuous conductive paths at lessened temperatures, making it advantageous in high-frequency electronic applications [56].

### 2.5. Thermal Conductivity

Silver is renowned for its remarkable thermal conductivity, which is around 429 W/mK at ambient temperature. This property allows silver to easily transmit heat [57]. Silver NPs possess exceptional heat conductivity due to their high surface-area-to-volume ratio, which remains consistent even at the nanoscale [58]. The main reason for the high thermal conductivity is mainly due to the efficient transfer of heat by phonons and electrons, which is helped by the small size of the particles. This reduces scattering and resistance at the grain boundaries [59]. Silver NPs offer superior thermal conductivity, reduced contact resistance, and oxidative resistance, making them suitable for various technological and industrial applications due to their lower sintered temperatures [60]. For example, Li et al. highlighted the efficiency of silver NPs in improving the thermal management of LED devices. Silver NPs were used in thermal interface materials to lower thermal resistance and improve heat dissipation from LEDs, thereby enhancing their performance and operational lifespan [61]. Ultimately, the exceptional thermal conductivity of silver NPs, together with their stability and straightforward manufacturing process, positions them as very promising instruments for enhancing heat management in future research efforts.

### 2.6. Optical Properties

Silver NPs have been considered extremely valuable due to their distinct optical properties. These properties are mainly associated with their LSPR, a phenomenon where conduction electrons on the NP surface oscillate in resonance with incident light [62]. This phenomenon results in the strong absorption and scattering of light, typically in the visible range. Consequently, silver NPs can demonstrate colors that can differ relative to their shape, size, and surrounding medium [63]. LSPR also results in enhanced electromagnetic fields near the NP surface. This makes silver NPs highly sensitive to environmental changes and enhances their use in various applications, including biological sensing, imaging, and photothermal therapy [64].

The increased refractive index sensitivity of silver NPs is also important for their utilization in sensor technologies [65]. Allowing for a more precise detection of changes in the surrounding medium, they improve the accuracy of the measurement. Also, the controlled manipulation of the NPs’ size, shape, and surface properties can significantly influence this sensitivity, enabling the optimization of their performance [66,67]. Furthermore, silver NPs are considered essential in Raman spectroscopy through a technique known as SERS [68]. SERS is a highly sensitive Raman spectroscopic technique that significantly enhances the Raman signals of molecules adsorbed onto the surface of silver NPs. In this manner, it allows for the detection of extremely low concentrations of Raman-active analytes, even at pico- and femtomolar levels [69].

In addition, a relationship was shown between optical properties and the antimicrobial efficiency of silver NPs. For example, Mlalila et al. demonstrated that silver NPs possessing specific surface plasmon resonance characteristics, such as narrow full width at half maximum and well-defined spherical shapes, tend to exhibit superior efficacy against various microbes, including bacterial strains such as *E. coli* [70].

In summary, the exceptional optical properties of silver NPs, characterized by their LSPR, result in distinctive light absorption and scattering characteristics. These properties not only make silver NPs suitable for a wide range of applications but also emphasize their crucial role in enhancing Raman spectroscopy through SERS. Considering these factors, silver NPs are likely to stand out in future research and applications, driven by their outstanding attributes.

### 2.7. Antibacterial Activity

Silver NPs exhibit significant antibacterial activity influenced by several variables including size, shape, surface charge, and the release of silver ions. The small size and large surface area of silver NPs facilitate their interaction with bacterial cell membranes, causing impairment of membrane integrity, release of cellular contents, and eventually cell death [71]. Although the antibacterial activity of silver NPs has not been thoroughly demonstrated yet, most of the proposed pathways are primarily associated with the release of silver ions.

Specifically, these ions interact with amine, phosphate, and thiol residues in enzymes and proteins, thereby disrupting their function and leading to cellular toxicity [72]. As an example, Li et al. demonstrated that silver ions released from NPs bind strongly to thiol groups present on bacterial membranes, resulting in the formation of complexes that disturb protein structures and enzymatic activities crucial for bacterial survival. Furthermore, it was stated that this process disrupts lipid–protein interactions within the bacterial cell membrane, leading to the leakage of intracellular substances and compromising the cell’s ability to maintain homeostasis and defend against environmental stresses [73]. Silver ions can generate ROS, inducing oxidative stress, hence impairing cellular components such as lipids, proteins, and DNA. Park et al. highlighted this by inspecting the antibacterial effects of silver ions released from silver NPs and their ability to generate ROS. In particular, they exposed Gram-positive and Gram-negative bacteria to silver ions and subsequently measured the levels of ROS. Under aerobic conditions, 2.2 and 3.3 log reduction in *E. coli* cell population was observed when 0.5 and 1.0 mg/L silver ions were administered, respectively. However, anaerobic conditions were less efficient, with 0.5 log and 1.9 log reductions for the same concentrations [74]. Beyond ion release, silver NPs exert bactericidal effects by directly affecting bacterial cell membranes. As they accumulate in surface pits, their small size causes membrane denaturation and structural changes. Thus, they disrupt organelles and lead to cell lysis [75]. Additionally, silver NPs interfere with bacterial signal transduction pathways, affecting protein phosphorylation and leading to cell apoptosis and the inhibition of cell growth [76].

**Figure 4:**
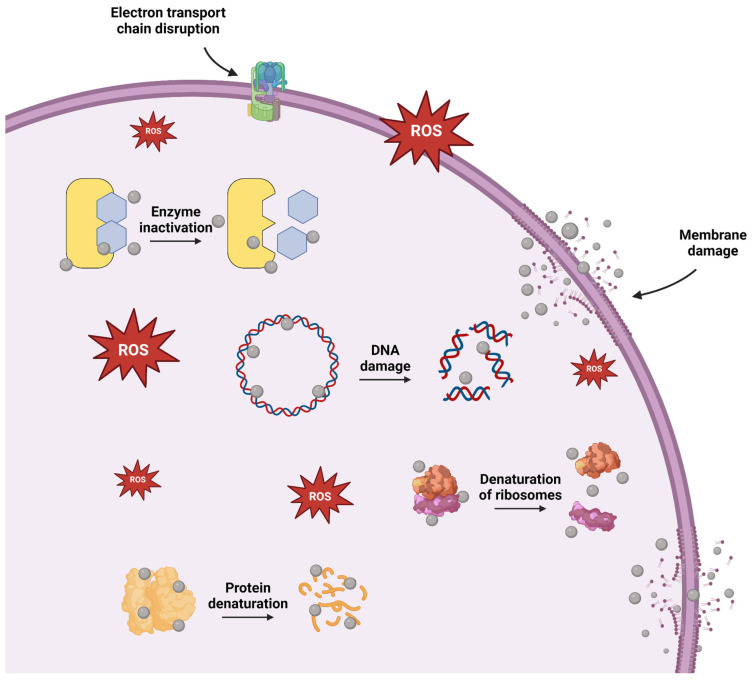
Illustration of the antibacterial mechanisms of silver NPs, including ROS-dependent pathways, DNA damage, enzyme inactivation, protein and ribosome denaturation, membrane damage, and electron transport chain disruption [85].

As a whole, these diverse mechanisms, including interference with cellular processes, highlight silver NPs as versatile and effective antimicrobial agents (Figure 4). Thus, they effectively target a variety of pathogens, such as common bacterial strains, including those that are both Gram-positive and Gram-negative. This capability of silver NPs to target a broad spectrum of pathogens is utilized in various applications (Table 1). For instance, silver NPs are used in bone graft materials and implants to leverage their dual benefits of antimicrobial action and bone healing promotion. When incorporated into bone grafts or implant coatings, silver NPs create an unfavorable environment for microbes, minimizing the chances of post-operative infections. Additionally, silver NPs contribute to bone healing by stimulating osteoblast activity and promoting mineralization at the application sites [77]. Also, silver NPs are increasingly employed in dentistry since they possess significant bactericidal effectiveness and antibiofilm activity, which can improve oral hygiene. Their application in dental materials and coatings helps to mitigate the possibility of bacterial infections associated with dental procedures. With that, they are positioned as essential components to improve oral health [6]. Similarly, antibacterial materials are extremely important in food packaging applications. Thanks to their strong antibacterial activity, silver NPs are currently used as a packaging material in many films and nanocomposites for food packaging applications [78].

**Table 1:** Applications of silver NPs as antibacterial agents.

Highlighted Property	Result	Reference
Antibacterial activity	Silver NPs synthesized with *Prosopis fracta* extract exhibit concentration-dependent antibacterial activity against *S. mutans*. MIC values are determined as 6.25 µg/mL, 12.5 µg/mL, and 100 µg/mL for NP concentrations of 1 mM, 3 mM, and 5 mM, respectively.	[79]
Antibacterial activity	Silver NPs with a size of 15 nm are utilized to sterilize bacteria present in water. Demonstrating a dose-dependent antibacterial effect, these NPs achieve 99.72% of bacterial inhibition. Optimum conditions are determined as pH 6 and 20 min of contact time at a concentration of 0.01 mg/mL.	[80]
Antibacterial activity	Antibacterial activity of the silver NPs synthesized from *Carduus crispus* are tested on both Gram-positive (*Micrococcus luteus*) and Gram-negative (*E. coli*) bacteria. Results reveal a size-dependent antibacterial activity. Thirteen nm of silver NPs demonstrat inhibition zones of 7.5 ± 0.3 mm against *M. luteus* and 6.5 ± 0.3 mm against *E. coli* in comparison to their larger counterparts.	[81]
Antibacterial activity	Spherical silver NPs with an average size of 20 nm are synthesized using *Cestrum nocturnum.* Bactericidal activity is evaluated on *Citrobacter*, *Salmonella typhi*, *Enterococcus faecalis*, *E. coli*, *Proteus vulgaris*, and *Vibrio choleraecitrobacter.* Maximum zone of inhibition observed is 41 mm against *V. cholera*, while the minimum is 15 mm against *E. faecalis.* MIC values are 16 μg/mL for *Citrobacter*, *S. typhi*, and *V. cholerae*; 8 μg/mL for *E. coli* and *P. vulgaris*; and 4 μg/mL for *E. faecalis* These results highlight silver NPs as promising alternatives to overcome antibiotic resistance and develop new antibiotic products.	[82]
Antibacterial activity	Spherical silver NPs, with sizes varying from 15 to 25 nm, are synthesized from cell-free beef extracts. NPs exhibit potent antibacterial activity against multidrug-resistant strains of *E. coli* and *S. aureus* (with a MIC of 40 µg/mL). Upon exposure to 50 µg/mL silver NPs, 97.5% reduction in colony-forming unit (CFU) values for *E. coli* and 96.7% reduction in *S. aureus* are observed. This novel approach represents a high potential for surface decontamination, and it is expected to significantly advance the development of disinfectants, surface treatment products, and nanomedicines containing silver NPs.	[83]
Antibacterial activity	Spherical silver NPs are synthesized using *Solanum nigrum* and *Indigofera tinctoria* extracts. Antibacterial activity of these NPs is evaluated using various concentrations (50 µL, 100 µL, 150 µL). All tested concentrations effectively inhibit the growth of bacterial pathogens including *Pseudomonas* sp., *S. aureus*, and *S. mutans.* Given these results, silver NPs are highlighted as effective coating materials for the development of surgical sutures, which possess minimal risk to humans and the environment.	[84]

These mechanisms can be influenced by multiple variables, such as size, shape, surface charge, and surface functionalization. Thereby, they collectively determine how effectively silver NPs interact with bacterial cells.

#### 2.7.1. Effect of Size on Antibacterial Activity

The antibacterial effectiveness of silver NPs is notably influenced by their size. Since smaller NPs exhibit a higher surface-area-to-volume ratio, it leads to an enhancement in factors, such as the release rate of silver ions and interaction with bacterial cells that are associated with bactericidal activity [76]. Smaller NPs also penetrate bacterial cell walls and membranes more easily, which leads to effective internalization and disruption of cellular functions. In addition, the rapid release of silver ions from smaller NPs interacts with bacterial proteins and enzymes, eventually causing cellular breakdown. Supporting this, researchers observed that approximately 81% of the NPs were released in a period of 48 h, with smaller ones showing greater release kinetics compared to their larger counterparts [86]. Furthermore, it was demonstrated that smaller silver NPs enhance the generation of ROS and contribute to oxidative stress. Analysis of NPs with different sizes, 1 nm and 70 nm, revealed that smaller NPs induced ROS more rapidly, within just 5 min, whereas larger NPs required 60 min to achieve similar results. Additionally, 1 h exposure to these NPs (at a concentration of 5 µg/mL) led to 40% reduction in cell viability of 70 nm silver NPs, while 1 nm of NPs resulted in complete (100%) cellular destruction [87].

#### 2.7.2. Effect of Shape on Antibacterial Activity

The effect of NP shape on antibacterial activity is currently highlighted in the literature. Specifically, since spherical NPs can efficiently release silver ions, they are widely employed in antibacterial research compared to their counterparts [88]. Additionally, the sharp edges and vertices of triangular NPs enable enhanced interactions with bacterial membranes, facilitating better penetration and disruption compared to the smoother surfaces of spherical NPs. At a concentration of 4 μg/mL, triangular silver nanoprisms exhibited larger zones of inhibition against *E. coli* (with a concentration of 10^5^ to 10^7^ CFU/mL), indicating greater bactericidal efficiency compared to spherical ones [89].

#### 2.7.3. Effect of Surface Charge on Antibacterial Activity

Modifying the surface charge is also a critical factor in defining the antibacterial activity of silver NPs. As previously stated, silver NPs with a positive charge have demonstrated greater antibacterial effectiveness as compared to NPs with a neutral or negative charge [44]. This property extends to a wide range of pathogens, including both antibiotic-resistant and non-resistant bacteria. To exemplify this, positively charged silver NPs can inhibit the proliferation of methicillin-resistant *S. aureus* (MRSA, TCH1516), as well as methicillin-sensitive *S. aureus* (TCH1516), with a MIC of 12.5 ppm [90]. This is mainly due to improved electrostatic contact between positively charged NPs and bacterial cell membranes leading to stronger adhesion and higher effectiveness in eliminating bacteria [91].

#### 2.7.4. Effect of Surface Functionalization on Antibacterial Activity

The antibacterial activity of silver NPs is highly influenced by their stability and dispersibility in biological environments. Therefore, functionalization of the NPs surface with biocompatible polymers, such as polyethylene glycol (PEG) or chitosan, is considered an effective approach to enhance stability and minimize aggregation [92,93]. For instance, PEG-functionalized silver NPs have been shown to exhibit an improved zone of inhibition against *S. aureus*, reaching 29 mm at pH 10, while bare silver NPs maintained a maximum zone of inhibition of 22 mm [92]. The functionalization of silver NPs not only prevents their aggregation but also extends their circulation duration and maintains their structural integrity in complex biological fluids. This avoids premature disintegration and enhances the antibacterial activity of the NPs [71].

Gaining knowledge about the variables associated with form, size, surface charge, surface functionalization, and silver ion release is essential to improve the efficiency of silver NPs in antibacterial environments.

### 2.8. Antifungal Activity

Silver NPs are regarded as potential antifungal agents, providing a new perspective on antifungal treatment (Table 2) [94]. Similar to antibacterial activity, the fungicidal effectiveness of silver NPs is attributed to multiple mechanisms. The main one is the formation of ROS, which causes oxidative damage to the cellular components of fungi. Additionally, silver NPs can disrupt fungal cell membranes, leading to increased permeability and eventual cell lysis [95].

Researchers highlighted silver NPs’ fungicidal activity against various species, including *Candida*, *Aspergillus*, and Fusarium. Their findings revealed the broad-spectrum antifungal activity of silver NPs against all tested fungi, with MICs ranging from 0.125 to 4.00 µg/mL. This efficiency surpasses that of conventional antifungal agents, such as fluconazole, which had MIC values ranging from 0.250 to 16.00 µg/mL and was effective only against the *Candida* species [96]. Moreover, Panáček et al. demonstrated silver NPs’ notable antifungal activity against pathogenic *Candida* spp. at concentrations around 1 mg/L of silver. It was stated that both silver NPs and ionic silvers exhibited efficient fungicidal activity, with silver NPs achieving a MIC value as low as 0.052 mg/L against *C. albicans* I. Also, both inhibited yeast growth similar to conventional antifungal agents without causing cytotoxicity in human fibroblasts [97].

Furthermore, the antifungal activity of silver NPs is known to be dependent on their surface modification. Matras et al. investigated the antifungal characteristics of silver NPs against phytopathogens, focusing on *Fusarium avenaceum* and *Fusarium equiseti*. Results indicated that although both silver NPs (positively- and negatively-charged), demonstrate strong antifungal effects, those carrying a positive charge exhibited superior efficacy, particularly at a concentration of 10 mg L^−1^. These results highlight the role of the surface chemistry of silver NPs in fungicidal activity against phytopathogens, suggesting promising avenues for further research [98].

**Table 2:** Applications of silver NPs as antifungal agents.

Highlighted Property	Result	Reference
Antifungal activity	Spherical silver NPs, with sizes varying from 3 to 13 nm, are synthesized using *Nigrospora oryzae.* Efficiency of different concentrations (50, 100, 150, and 200 ppm) of silver NPs are evaluated on *Fusarium* spp. All concentrations are able to inhibit fungal growth, with higher concentrations resulting in greater inhibition. Findings highlight silver NPs as potent antifungal agents in the field of agriculture since they are capable of replacing synthetic chemicals used to control fungal pathogens.	[99]
Antifungal activity	Silver NPs are synthesized using different concentrations of SDS (25 and 50 mg) as a reducing agent. NPs containing 50 mg SDS (Silver NP-50) show greater antifungal activity against *Candida parapsilosisi* Further, an antifungal cream is developed incorporating the silver NP-0.50 formulation with miconazole, a fungicidal agent, to combine its effects and enhance therapeutic efficiency against fungal infections.	[100]
Antifungal activity	*Ageratum conyzoides* leaf extracts are used to synthesize silver NPs. Silver NPs are incorporated into fabrics, which are then tested for their antifungal capability against *Aspergillus* sp. Findings highlight the fungicidal effectiveness of silver NPs on the development of antifungal textiles as demonstrated by their maintained efficiency even after five washing cycles.	[101]
Antifungal activity	Cellulose-based films containing different concentrations of silver NPs (0.10%, 0.25%, 0.50%) are produced. Addition of silver NPs into films causes enhanced antifungal activity, with 0.25% silver NPs showing effective fungicidal properties against *Colletotrichum gloeosporioides*. Results lead to the development of effective fruit coatings, which prevent fungal growth after 14 days of storage while preserving the fruit’s quality.	[102]

### 2.9. Antiviral Activity

Silver NPs show antiviral properties characterized by their ability to interact effectively with viruses, which make them appropriate materials for various applications (Table 3). Although the exact mechanism has not been fully elucidated yet, it is mostly attributed to their capability of binding to viral proteins and disrupting viral envelopes [103]. With that, they prevent the virus from attaching to and entering host cells, and they block the viral replication as well as the formation of new viruses. For example, research has established that silver NPs can prevent the replication of various viruses [104]. Silver NPs are considered alternative antiviral agents against the influenza A virus, owing to their high efficiency at both pre- and post-treatment of MDCK cells. These NPs demonstrate strong virucidal effects, particularly at concentrations ranging from 4 to 100 μg/mL, where the inhibition of viral cytopathic effect ranges between 82% to 100% [103]. Similarly, silver NPs are also being investigated for their potential against SARS-CoV-2 in VeroE6/TMPRSS2 cells. PVP-coated silver NPs, specifically those having a diameter around 10 nm, are capable of effectively blocking viral replication. Data from the plaque assays confirm that these NPs achieve complete inhibition (100%) at a 0.05 multiplicity of infection (MOI) and possess partial inhibition at higher MOIs (0.5) [105].

Also, it is crucial to consider that the effectiveness of silver NPs in antiviral research is strongly dependent on their synthesis parameters, such as size, shape, and surface functionalization [106]. Specifically, smaller NPs featuring larger surface areas tend to interact effectively with viral particles and exert virucidal effects. Regarding these, Elechiguerra et al. demonstrated that smaller silver NPs, those less than 14 nm in diameter, exhibited preferential binding to the gp120 glycoprotein knobs on the HIV-1 viral envelope. In contrast, larger NPs, with diameters exceeding 10 nm, were not observed to effectively bind to these specific sites [107].

In addition, surface functionalization plays a key role in determining the antiviral efficacy of silver NPs. Specifically, functionalizing the NPs with specific ligands or biocompatible polymers enhances their stability, prevents aggregation, and enables them to target specific viral proteins or receptors [108]. To sum up, the surface properties of silver NPs can be manipulated to increase selectivity and potency of their antiviral activity while minimizing the potential for off-target effects and reducing the toxicity to host cells at the same time. Although further research is required to optimize silver NPs utilization in antiviral studies, they are distinguished as important materials for the development of new therapeutic strategies.

**Table 3:** Applications of silver NPs as antiviral agents.

Highlighted Property	Result	Reference
Antiviral Activity	Silver NPs are synthesized using collagen to evaluate their virucidal activity against SARS-CoV-2. *In vitro* studies demonstrate silver NPs’ dose-dependent inhibitory effect on SARS-CoV-2, which leads to development of mouthwash and nasal rinse formulations containing silver NPs. These formulations’ efficiency is tested in a clinical trial that results in a significantly lower SARS-CoV-2 infection rate (1.8%) in the experimental group compared to (28.2%) the control group.	[109]
Antiviral activity	Silver NPs are synthesized via the reactive blade coating (RC) method to assess their antiviral properties against HCoV-229E. Then, an RC-silver NP coating is applied to personal protective equipment (PPE), including glass, face masks, and cotton textiles, to test its efficiency. Findings reveal that the RC-silver NP coating enhances the virucidal properties of PPE, achieving up to 99.9% reduction in viral activity after 30 min of exposure.	[110]
Antiviral activity	Spherical NPs are produced from different silver nitrate (AgNO_3_) solutions, 50, 100, 150, and 200 mM, to develop an active packaging material, a paper, coated with silver NPs. NPs’ antiviral properties are then tested on Dengue virus serotype 3 (DENV-3) strain P12/08. The paper coated with silver NPs (prepared from the 150 mM AgNO_3_ solution) demonstrates complete inactivation (100%) of DENV-3 within one minute of exposure.	[111]
Antiviral activity	Antiviral paint is developed to reduce the hazards from contaminated high-touch surfaces. *Saccharum officinarum* leaf extract is utilized to synthesize silver NPs with an average size of 11.7 ± 2.8 nm. Further, synthesized NPs are incorporated into architectural paints and subsequently evaluated for their virucidal efficiency against human coronavirus NL63. Paint containing 80 ppm silver NPs exhibits significant antiviral activity, achieving over a 90% reduction in comparison to the untreated control.	[112]

### 2.10. Anticancer Activity

The use of nanomaterials has drawn great interest in anticancer research. In this context, silver NPs are one of the major NPs that are used in anticancer research due to their distinct physicochemical properties (Table 4) [113]. Silver NPs demonstrate anticancer activity through multiple mechanisms. Induction of oxidative stress through ROS generation is one of the major mechanisms behind the anticancer activity of silver NPs. Increased levels of oxidative stress damage structural components, such as DNA, proteins, and lipids, inducing apoptosis and autophagy. It was shown that biosynthesized spherical silver NPs (with a concentration of 150 µg/mL) increased ROS generation 2.4- and 1.7-fold in comparison to the control group, following 24 and 12 h administration. Additionally, an 18% and 24% increase in the number of early and late apoptotic cells were observed, depending on the concentration (50 µg/mL and 150 µg/mL, respectively) [114]. In addition, silver NPs can disrupt the mitochondrial function, causing ROS production and mitochondrial-mediated apoptosis. Mitochondrial disruption can be evidenced by a decrease in the mitochondrial membrane potential, as indicated by a shift from red to green fluorescence in JC-1 staining (a fluorescent dye used to assess mitochondrial health). These align with the increase in ROS production, where a significant rise in fluorescence intensity, from 146 to 341, is observed in cells treated with silver NPs for 3 h. Furthermore, this disruption leads to the release of cytochrome C from the mitochondria into the cytosol and activates caspase enzymes to break down the cell [115]. In addition, silver NPs have the ability to disrupt several cellular signaling pathways, such as MAPK and PI3K/Akt, which are considered important for cell survival and proliferation. This disruption eventually leads to decreased cancer cell viability and increased apoptosis [116].

Autophagy modulation is another crucial mechanism behind the anticancer activity of silver NPs. Thus, it was indicated that silver NPs can disrupt autophagic flux, causing the accumulation of autophagosomes and inducing cell death [117]. Moreover, silver NPs can inhibit angiogenesis, and the formation of new blood vessels, which is necessary for tumor expansion. Those NPs have been shown to limit the growth and dissemination of tumors since they effectively block the blood flow through targeting endothelial cells and disrupting pro-angiogenic signaling molecules [116].

In conclusion, silver NPs are considered as having promising anticancer effects through various mechanisms, such as inducing oxidative stress, disrupting cellular signaling pathways, impairing mitochondrial function, and modulating autophagy and angiogenesis. These actions collectively contribute to reducing cancer cell viability and inhibiting tumor growth, offering the utilization of silver NPs as a potential therapeutic approach in oncology. Nevertheless, further research is required to fully understand their specific interactions, long-term effects, and safety profiles.

**Table 4:** Applications of silver NPs in cancer research.

Highlighted Property	Result	Reference
Anticancer Activity	Researchers develop bovine serum albumin (BSA) coated spherical silver NPs as effective photothermal therapy (PTT) agents in treating skin cancer. BSA-coated silver NPs effectively convert laser light into heat, depending on the NP concentration and laser power, which further leads to significant reduction in B16F10 melanoma cells. These indicate the importance of developing silver NP-integrated PTT formulations for cancer treatment.	[118]
Anticancer activity	Oval PG-Silver-PPa nanoconjugates (NCs) with an average diameter of 61.9 mm are synthesized for enhanced photodynamic therapy (PDT) in treating cancer. NCs’ effectiveness in PDT is tested on Eca-109 cancer cells. Results reveal NCs’ superior performance, evidenced by increased cellular uptake and higher singlet oxygen generation compared to precursor drug PPa alone. These findings suggest that PG-Silver-PPa NCs have the capability of serving as potential alternative PDT agents.	[119]
Anticancer activity	Spherical silver NPs, with sizes around 13 ± 1 nm, are synthesized using a chemical solution method. Effectiveness of silver NPs, with varying concentrations (2, 5, 10, 25, 50, 100, and 200 µg/mL) are examined on HepG2 and MCF-7 cancer cell lines. All concentrations of silver NPs induce cytotoxic effects, with higher concentrations resulting in greater cell death. Findings underscore the silver NPs’ potential as effective nanodrugs in emerging cancer research.	[120]
Anticancer activity	*Dictyota ciliolata* extract is used to synthesize spherical silver NPs with an average particle size of 100 nm. Activity of silver NPs are tested at different concentrations (10, 20, 30, and 40 µg/mL) on A549 lung adenocarcinoma cells. NPs are successful in inhibiting the cancer cell proliferation as well as reducing tertiary capillary formation. These underline silver NPs’ antiangiogenic properties and highlight their potential as promising agents in the treatment of lung cancer.	[121]

### 2.11. Anti-Inflammatory Activity

Silver NPs possess notable anti-inflammatory properties that makes them valuable in various biomedical applications. These NPs are capable of reducing inflammation by modulating the activity of inflammatory cells and inhibiting the release of pro-inflammatory cytokines. In this way, they help control the body’s inflammatory response [19]. Significant reduction of inflammation is primarily desired in biomedical applications, especially in wound healing and bone repairs, since controlled inflammation is an important factor in a successful recovery process. For instance, silver NPs are considered an effective material for wound healing applications because of their unique anti-inflammatory and antimicrobial characteristics. Thanks to their wide-ranging antimicrobial activity, silver NPs can effectively prevent infection in wounds (Table 5) [71]. Based on this, silver NPs are also used in bone graft materials and implants for their antimicrobial activity and bone-healing promotion. When incorporated into bone grafts and implant coatings, silver NPs minimize the chances of post-operative infections through negatively influencing the environment for microbes. In addition, silver NPs contribute to bone healing by stimulating osteoblast activity and promoting mineralization at the application sites [77].

In addition, silver NPs promote tissue regeneration by stimulating both cell proliferation and migration, thus increasing the rate of wound closure. The excessive inflammation that hinders the healing phase and leads to chronic wounds can be reduced by the silver NPs’ anti-inflammatory properties [4].

Overall, these attributes establish silver NPs as a valuable tool in advanced wound care, offering a comprehensive approach to accelerate recovery and improve the effectiveness of the healing process.

**Table 5:** Applications of silver NPs as anti-inflammatory agents.

Highlighted Property	Result	Reference
Anti-inflammatory activity	Spherical silver NPs with an average size of 25.92 nm are incorporated into riclin-based hydrogels. Anti-inflammatory activity of nanocomposite hydrogels are assessed by analyzing the expression of pro-inflammatory cytokines (IL-1α, IL-6, and TNF-α). Wounds treated with riclin-silver NP composite have considerably lower levels of IL-1α, IL-6, and TNF-α compared to controls, indicating reduced inflammation. These suggest that silver NPs have promising potential to be utilized in wound dressings.	[122]
Anti-inflammatory activity	Collagen-based hybrid biomaterials containing silver NPs, with sizes 30 to 50 nm, are synthesized. Anti-inflammatory efficiency of the biomaterials is evaluated measuring the secretion of pro-inflammatory cytokines, IL-6, IL-1β, and TNF-α. A significant reduction in the secretion of these cytokines are observed, which is attributed to the presence of silver NPs. Results indicate these hybrid scaffolds are strong anti-inflammatory agents, with potential applicability in periodontal disease treatment.	[123]
Anti-inflammatory activity	Silver NPs synthesized from different extracts of *Ehretia cymosa* (methanol, n-hexane, and ethyl acetate) are included in cream formulations. Anti-inflammatory activity of these creams is measured by carrageenan-induced rat paw edema method on albino rats. Creams demonstrate a significant reduction in inflammation, specifically those containing the NPs synthesized from ethyl acetate, which achieves a 100% anti-inflammatory effect within 4 h.	[124]
Anti-inflammatory activity	Spherical silver NPs, with sizes ranging from 30.99 to 68.20 nm, are synthesized using aqueous curcumin extract. Anti-inflammatory effects of silver NPs are tested in a rat model of adjuvant arthritis at a concentration of 100 mg/kg. Silver NPs reduces the levels of inflammatory markers (IL-6 and hs-CRP) and paw edema in arthritic rats. These findings position silver NPs as highly effective candidates for developing anti-inflammatory drugs.	[125]

## 3. Synthesis of Silver Nanoparticles

Silver NPs, due to their remarkable stability and low chemical reactivity relative to other metals, constitute a noteworthy advancement in the field of nanotechnology. Their distinct physicochemical characteristics have generated significant attention for a range of biological uses. In addition, they can be produced utilizing several approaches, such as chemical, biological, and physical procedures [13]. For the synthesis of silver NPs, conventional methods, such as chemical and physical procedures, are commonly employed. However, these methods come with several disadvantages, including the use of hazardous reactants, complex purification processes, lower conversion efficiency, unstable yields, environmental risks, high energy consumption, and increased costs [126]. In comparison, NPs produced through biological methods are significantly superior. There is a continuous need for the development of synthesis approaches that are economical, cost-effective, non-toxic, and productive to eliminate hazardous waste [127]. Plants, algae, and microbes are all used in biological methods. However, the microbial approach has drawbacks, including longer processing times and challenging steps in maintaining cultures [14].

**Figure 5:**
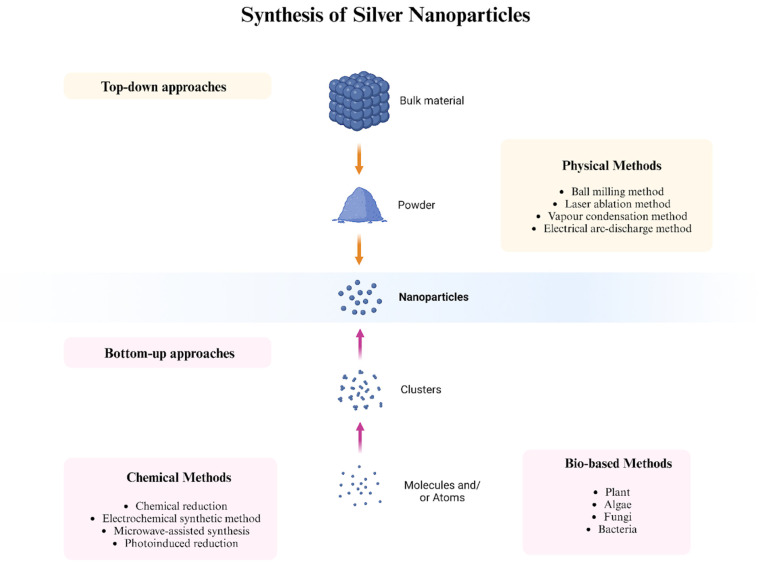
A top-down and bottom-up method to the synthesis of silver NPs. NPs are generated from bulk materials in the top-down technique and from molecular components in the bottom-up strategy [128].

Top-down and bottom-up techniques are the two fundamental approaches used for synthesizing NPs (Figure 5). These strategies encompass three main synthesis methods: physical, biological, and chemical. The physical technique utilizes a top-down approach, whereas the biological and chemical methods generally rely on a bottom-up strategy [15]. The top-down method employs physical processes, such as electrical arc discharge, vapor condensation, grinding, milling, and laser ablation, to convert bulk materials into NPs. These techniques can produce NPs with typical sizes ranging from 10 to 100 nm without requiring chemical additives. Physically synthesized NPs often exhibit high purity and uniform size. However, a notable drawback of this method is the lack of stabilizers or capping agents, which are crucial for preventing agglomeration as no potentially hazardous chemicals are involved [85]. In a bottom-up process, atoms and molecules aggregate to form NPs from smaller building blocks. The following are a few examples of bottom-up techniques: chemical vapor deposition, laser pyrolysis, sol-gel processing, bioassisted synthesis, self-assembly of monomer molecules, plasma or flame spraying, and electrochemical or chemical nano-structural precipitation [16].

### 3.1. Physical Methods

Silver NPs are physically synthesized using both vapor-based and mechanical methods. Particle-size reduction involves the employment of several energies, such as thermal energy (physical vapor deposition) [129], light energy (laser ablation method) [130], electrical energy (electrical arc-discharge method) [131], and mechanical energy (ball milling method) (Table 6) [132].

#### 3.1.1. Ball Milling Method

The ball milling process, also known as mechanical ball milling, is a classic method frequently used to manufacture silver NPs in a solid state. The process of ball milling involves the application of kinetic energy from moving balls to the material to fracture material particles and disrupt chemical bonds, creating new surfaces. Usually, the newly formed surfaces’ dangling bonds are chemically reactive. Furthermore, it has been shown that the application of a high-energy ball milling process can occasionally result in localized high pressure of several GPa and/or a temperature above 1000 °C. Because of the nature of the ball milling process, it has been utilized to create novel nanostructures with novel chemical characteristics through mechano-chemical synthesis [133] The mechanical ball milling technique involves placing metal components, along with a specified mass ratio of milling balls and an inert gas or air, into a rotating container. The morphology of the metal materials is significantly influenced by the milling time, rotational speed, and type of atmospheric medium used during the milling process [132,133]. Because of this feature, the ball milling process has been used as a mechano-chemical synthesis method to produce unique nanostructures with cutting-edge chemical properties [133].

Planetary ball milling was used in a mechanochemical process by Khayati et al. by including organic process control agents (PCAs) [134]. The study employed various types of PCA to determine particle sizes, which ranged from 14 to 34 nm, and it observed their crystallite shapes. In a different study, PEG was used as the stabilizing agent in the mechanochemical ball milling process, resulting in silver NP crystallites with an average size of 10–12 nm [135]. Effective antibacterial action against both Gram-positive and Gram-negative bacteria was made possible by the small size of the silver NPs that were produced. Ball milling is a cost-effective method that produces silver NPs in a solid form at room temperature with reasonable control over particle size [136].

#### 3.1.2. Laser Ablation Method

Laser ablation is a process where a pulsed laser is used to rapidly heat a sizable metal target that is submerged in water or an organic solvent, leading to the creation of a plasma plume. As the plasma plume loses heat, tiny particles of metal begin to form and increase in size, eventually becoming clusters at the nanoscale level [137]. NPs are capable of absorbing photons during laser ablation through processes such as multiphoton absorption, interband transitions, and plasmon excitations. The processes are directly influenced by characteristics such as laser fluence, wavelength, and pulse duration. Furthermore, the characteristics of the NPs can be altered by the specific type of aqueous solution employed [130].

Silver NPs produced using this technique are kept extremely pure because they do not contain any ligands or chemical stabilizers, which gives them special surface properties [130]. As a result, this approach provides a safe biological substitute for techniques that are usually needed for chemical stabilizers, which makes it appropriate for industries such as food and medicine. Additionally, this approach can produce modest NP sizes, with low agglomeration rates and narrow size distributions [138,139]. Laser ablation offers several benefits, but certain drawbacks limit its practical application. The process is generally not very productive and poses challenges for industrial-scale use. High-energy lasers are required to achieve the desired concentrations, which significantly increases the cost [139].

#### 3.1.3. Vapor Condensation Method

Two broad categories can be used to classify the fundamental and most often utilized physical vapor deposition processes: arc sputtering and evaporation. In arc sputtering, plasma is produced by highly ionized metal vapor. In evaporation, the target coating material is bombarded with a high-energy electrical charge to deposit metal on the substrate. Atoms are mechanically ejected from the target material during this process by impact from ions and energetic atoms [140]. To sum up, the physical vapor deposition technique may produce pure, dispersible silver NPs with small particle sizes, but it requires complex equipment and outside energy.

#### 3.1.4. Electrical Arc-Discharge Method

Another well-liked physical method for producing silver NPs is the arc discharge process. Using this procedure, a high-current DC circuit with a cathode and an anode connected is run through a solvent, often deionized water [141]. Electrodes can be made from an inert metal, such as titanium, or from the metal of interest, namely silver, which will form the NPs [142]. An electric discharge between the cathode and anode of titanium electrodes utilizing AgNO_3_ as the precursor results in electron exchange in the plasma zone where silver ions are reduced. If silver electrodes are used, the silver melts and evaporates from the electrode ends, forming condensates that eventually develop into NPs [141].

The arc discharge process has several benefits, including fewer manufacturing steps, minimal impurity because it only requires the use of water, and simpler apparatus and equipment. Additionally, this technique can quickly achieve high NP synthesis rates. The NPs generated have a relatively broad size distribution in comparison to NPs produced by chemical techniques, but the size distribution is still large [142,143].

### 3.2. Chemical Methods

Chemical synthesis continues to be the most widely used process to produce silver NPs. Under specific conditions, silver NPs are synthesized by electron transfer from Ag^+^ in a precursor silver salt [144]. To balance safety and efficiency, complex methods for synthesizing silver NPs have been developed to optimize these processes. Innovations include the use of eco-friendly stabilizers and reducing agents, which enhance the biocompatibility of the NPs and minimize hazardous byproducts. These techniques are continually being refined to lower production costs and increase scalability, thereby expanding potential applications in various industries, such as electronics and medical equipment (Table 6) [145,146].

Typically, these agents are surfactants with protective properties, particularly polymeric compounds. They coat silver NPs, shielding their surfaces from further aggregation by preventing additional silver NPs from adhering to or attaching to them [147].

#### 3.2.1. Chemical Reduction

A variety of methods, such as the polyol approach, the chemical reduction method, and the radiolytic process, have been developed for the chemical synthesis of silver NPs. The most straightforward and effective way to produce silver NPs without aggregation is chemical reduction [148]. Reducing agents, stabilizers, and salt precursors are the three parts of the chemical reduction technique. Various reducing agents can efficiently reduce silver precursors to silver NPs when a stabilizer is present. Alternative silver precursors, such as AgNO_3_ [149], silver ammonia (also known as Tollens reagent) [150], silver sulfate [151], and silver chlorate [152], are constantly supplying monomers for nucleation. Hydrazine, NaBH_4_, TSC, ascorbic acid, ethylene glycol, polysaccharides, and formaldehyde are examples of reducing compounds that are frequently used. The kinds and ratios of reducers and precursors, as well as the solution’s pH and temperature, can all affect the characteristics of silver NPs. Using the chemical reduction approach, silver NPs with various desired shapes—such as nanospheres, nanoprisms, nanoplates, nanowires, nanocubes, and nanorods—can be produced [153].

#### 3.2.2. Electrochemical Synthetic Method

The electrochemical technique can decrease Ag^+^ to Ag^0^ while producing an electric potential in the electrolyte [154]. Under the influence of an external electric field, silver NP nucleation and growth occur nearly simultaneously. Using the electrochemical approach, silver NPs of various sizes can be produced by varying the current density. Additionally, the choice of solvents, electrolytes, and electrode types plays a crucial role in the synthesis of size-controlled silver NPs. Longer implementation times, higher precursor concentrations, and stronger current intensities during the synthesis process will result in the production of more silver NPs with smaller sizes [155].

#### 3.2.3. Microwave-Assisted Synthesis

The field of microwave-assisted synthesis was initially introduced in the early 1940s. The process involves using microwave irradiation to quickly heat the silver precursor, which could aid in on-site nuclei formation. As a result, the regulation of silver NP synthesis nucleation and growth phases is enhanced. According to data, silver NPs made with microwave assistance exhibit a high degree of crystallization and a narrow size [156,157].

A number of factors could influence the synthesis of silver NPs using microwaves, including the kind and concentration of stabilizer, the dielectric constant, the refractive index of the medium, the chirality of the reducing agents, and the power input and duration of the microwave. In addition to its high energy conversion efficiency, timesaving, ease of use, and cleanliness, the microwave-assisted approach is particularly advantageous since it can produce high dispersive silver NPs on a wide scale [158].

#### 3.2.4. Photoinduced Reduction

Metal precursors are first subjected to a photocatalytic reduction process by a reducing agent, which transforms them from n+ valence (Mn+) to zero valence (M0). Nuclei, or nucleation centers, are produced by the M0 and multiply to form metallic NPs [159]. Examples of light sources include sunlight, laser light, and ultraviolet light, which is the most common type [85]. NPs can be produced by photochemical synthesis in a range of media, including glassware, emulsions, polymer films, cells, and surfactant micelles. The source, intensity, and wavelength of the light, as well as the duration of the irradiation, are some factors that can affect the synthesis of silver NPs. For example, it has been demonstrated that extending the time and intensity of irradiation encourages an Ag⁺ reduction [159].

#### 3.2.5. Microemulsion Techniques

The term “microemulsion techniques” describes the process of synthesizing silver NPs by dispersing two immiscible liquids, such as water and oil, water and surface CO_2_, or a combination of water, oil, and one or more surfactants [156]. Using this technique, homogenous silver NPs with regulated sizes can be produced [160].

Silver precursors and reducing agents are the reactants that are first spatially separated into two immiscible phases, which forms the basis for the synthesis of silver NPs in two-phase aqueous organic systems [161]. One can classify “ready-to-use” surfactants as nonionic, cationic, zwitterionic, or anionic agents. Sodium dodecylbenzene sulfonate (SDS), lauryl sodium sulfate, and bis(2-ethylhexyl) sulfosuccinate are a few examples of anionic surfactants. Examples of cationic surfactants include cetyltrimethylammonium bromide (CTAB) and polyvinylpyrrolidone (PVP). An example of a nonionic surfactant is Triton X-100 [162].

### 3.3. Bio-Based (Green-Synthesized) Methods

Silver NPs have been biologically synthesized by a range of microorganisms and plants in recent decades. Microorganisms have the potential to evolve genes that allow for metal tolerance and metal bioconcentration, enabling them to thrive in environments high in silver. One of these adaptive evolutionary strategies is to modify and reduce the cytotoxicity of metals, leading to the production of silver NPs (Table 6) [163,164].

#### 3.3.1. Plants

Compared to microorganism-based methods, plant-based synthesis of silver NPs is more extensively employed because it is more efficient, less bio-compromising, and does not require active cell cultures. Various plant parts can be used for synthesizing silver NPs, including bark, callus, flower, fruit, leaves, peel, rhizome, stem, and seed [85,165]. The morphologies of silver NPs derived from plants are typically spherical or oval [166].

Plant extracts are rich in carbohydrates, proteins, flavonoids, polyphenols, and enzymes. In the process of producing cell-free metallic NP, certain phytochemicals are extracted and used directly as reducing and stabilizing agents, taking the place of potentially hazardous materials, including sodium borohydride (NaBH_4_). The diverse range of phytochemicals in the extracts makes it difficult to identify the precise mechanism behind this activity. However, proteins, polyphenols, and organic acids are thought to be the main reducing agents, with additional phytochemicals contributing to the overall process. For large-scale production, this approach is commonly considered economical [167,168].

Plant-mediated silver NP synthesis is influenced by a number of reaction parameters, such as pH, temperature, length of the reaction, and the concentration of precursors and extracts from plants. Variations in the synthesis conditions can yield silver NPs with different sizes and shapes. Therefore, a range of reaction conditions can regulate the synthesis of silver NPs by plants. Additionally, distinct plant sections demonstrate varying capacities for silver NP production. This suggests that to modify the characteristics of silver NPs for specific applications, it is imperative to optimize these parameters. By understanding the impact of distinct plant components on silver NP synthesis, more effective and targeted production techniques can be developed, enhancing the potential applications of silver NPs in various industries, including materials science, environmental remediation, and medicine [169,170].

Because of their non-pathogenic nature, environmentally friendly reaction conditions, and extremely cost-effective one-step process, plant extracts have been found to be a faster method for synthesizing silver NPs by green synthesis than microorganisms such as bacteria and fungi [166]. Moreover, the bioactivity of silver NPs derived from plants appears to be higher than that of silver NPs produced chemically. The antioxidant activity of the two approaches was assessed using the DPPH (1,1-Diphenyl-2-picrylhydrazyl) test, as developed by Sreelekha et al., using *M. frondosa* leaf extract and sodium citrate. Green NPs demonstrated 91% scavenging activity at 5 μg/mL compared to 79% activity at the same concentration for chemically generated NPs. The flavonoids and phenolic compounds found on the surface of the plant-derived NPs contribute to their antioxidant properties, making them beneficial for the treatment and prevention of degenerative disorders [171]. Furthermore, U87 and HEK 293 cell lines had a notable response to silver NPs produced from *Brachychiton populneus*. As the concentration increases, the proportion of living cancer cells decreases [172].

In this sense, plant-mediated production of silver NPs using plant extracts is a feasible method due to its easy availability, nontoxicity, simplicity, cost-effectiveness, and high reduction potential.

#### 3.3.2. Algae

The production of silver NPs using marine-based microorganisms has emerged as one of the novel and promising strategies due to its non-toxicity and environmentally benign character [173,174]. Many organic substances that are physiologically active are found in algae, including pigments, proteins, carbohydrates, polysaccharides, enzymes, and secondary metabolites [175]. Algae are one of the most excellent choices for producing silver NPs because of their rich content containing organic compounds. These compounds can serve as reducing agents to manufacture silver NPs with controlled sizes and shapes, such as spheres, triangles, cubes, rods, wires, hexagons, and pentagons. Various types of algae, including those derived from Rhodophyceae, Phaeophyceae, Chlorophyceae, and Cyanophyceae classes, have shown the ability to synthesize silver NPs [176]. According to these findings, algae provide a valuable bioresource for synthesizing silver NPs in various sizes and shapes. Various biomolecules found in algal extracts, including proteins, amino acids, and sulfated polysaccharides, are capable of functioning as capping or stabilizing agents and assisting the creation of silver NPs with various properties [177]. Low reaction temperatures, the use of non-hazardous reagents, and the manufacture of very small particles with a homogenous morphology are only a few benefits of the algae-mediated synthesis of silver NPs. However, the primary drawback of this biosynthesis method is its notably low production rate [178,179].

Similar to photosynthesis, the synthesis of silver NPs mediated by algae is advantageous for both the environment and the economy [180]. Microalgae play a significant role in the synthesis of silver NPs due to their ability to accumulate and reduce heavy metal ions. Algae facilitate an environmentally friendly production process as they operate under normal pH levels, ambient temperature, and pressure. Thus, the overall synthesis process is considered “green”. The synthesis can be classified as either extracellular or intracellular, depending on whether the reaction occurs inside the cells or outside, influenced by the presence of biomolecules in the cell culture [181]. For instance, because the cell wall, which normally serves as a barrier, limits the migration of metal cations into the cytoplasm, cells without a cell wall are generally more prone to intracellular production of silver NPs [182]. The size of generated silver NPs varies depending on the kind of cell; nevertheless, silver NPs with typical diameters as low as 4.3 nm [183] and as high as 35 nm [184] have been described. Upon synthesis, the silver NPs are encapsulated by a matrix of polysaccharides entering and exiting the cells [181]. Dahoumane et al. also emphasized the function of polysaccharides in NP stability and the significance of algal respiratory enzymes in NP [185].

There are numerous current studies on the effective manufacture of silver NPs with various noteworthy medicinal characteristics utilizing algal extracts, particularly brown algae [186,187]. In conclusion, the production of silver NPs by the use of algae extracts offers an easy, long-lasting, and green method. Due to their substantial organic content, high metal accumulation capability, and quick development, algae are good options for producing silver NPs.

#### 3.3.3. Fungi

Silver NPs synthesis by fungal-assisted synthesis is a simple and efficient method [188,189]. Fungal-mediated synthesis can produce silver NPs either intracellularly or extracellularly, depending on whether mycelia or fungal cell-free filtrate is used. Intracellular synthesis generally yields smaller NPs and involves fewer purification stages, which is an advantage. However, extracellular synthesis is often preferred due to the ease of collection and post-synthesis processing, making it more convenient for practical applications [85]. Protease, cellulase, chitinase, glucosidase, and many other fungal proteins and enzymes can directly promote and speed up the synthesis of silver NPs. Fungi convert Ag^+^ ions to Ag^0^ by the action of NADH and NADH-dependent nitrate reductase. Compared to other fungi, filamentous fungi are more interesting because they produce silver NPs with stable morphological characteristics and a wide range of uses [156]. To acquire the appropriate NP features, variables including agitation, temperature, light, culture, and synthesis times can be changed according to the type of fungus being employed [190].

Previous research has demonstrated that an enzymatic mechanism occurs during fungal synthesis processes, which influences the production of stable silver NPs in the 5–15 nm range [191]. This range, however, may change depending on the conditions of the reaction. FTIR data indicates that, similar to bacteria, carbonyl, amide, and hydroxyl groups that correspond to the cellular protein generate and stabilize silver NPs [192]. Fungi appear to be more useful than bacteria due to their higher rate of synthesis and less non-pathogenic behavior. Silver NPs produced by fungi have demonstrated observable antibacterial action. Naqvi et al. demonstrated that biocidal efficacy against drug-resistant bacteria was greatly enhanced by producing silver NPs utilizing *Aspergillus flavus* [193]. Using *Cladosporium cladosporioides*, Balaji et al. synthesized silver NPs by extracellular manufacturing techniques [194]. The produced silver NPs had typical diameters that varied from 10 to 100 nm. *Aspergillus terreus* was used to create spherical-shaped, 1–20 nm-sized silver NPs [195]. According to Mukherjee et al., a green method was used to produce monodispersed silver NPs using the fungus Verticillium [196].

In summary, the synthesis of silver NPs mediated by fungi is a practical, efficient, economical, and energy-saving biological approach. However, to ensure the safety of the final products, it is important to address and minimize any potential contamination on the surface of the silver NPs.

#### 3.3.4. Bacteria

Despite the challenges associated with NP synthesis—such as complex methods, difficulties in controlling NP size, and risks of culture contamination—bacteria remain a highly desirable resource. Microbes’ remarkable capacity to lower heavy metal ions makes them one of the most promising possibilities for NP synthesis [166,197].

Numerous investigations have documented the use of microorganisms in the manufacture of silver NPs. Using the *Pseudomonas stutzeri* strain, Klaus et al. reported synthesizing silver NPs with distinct compositions and morphologies. One of the first investigations to use microbes to produce silver NPs was this one [163]. Using psychrophilic bacterial culture supernatants, Shivaji and Madhu synthesized silver NPs [198]. Additionally, Nanda and Sravanan investigated the use of *S. aureus* in the manufacture of silver NPs [199]. The synthesis of silver NPs using *Bacillus licheniformis* biomass was previously described in a work by Kalimuthu et al. According to reports, an enzyme called nitrate was used to stabilize the synthesized NPs, which ranged in size from 40 to 50 nm [200].

The capacity of bacteria to endure in an exceptionally silver-rich environment could be a factor in the build-up of silver NPs [163]. The extracellular approach is better than the intracellular method since silver NPs are easier to recover [85]. In bacteria, organic compounds, such as peptides, reductase, cofactors, c-type cytochromes, and silver-resistant genes, can function as reducing agents. *S. aureus* [199], *Rhodococcus*, *Brevundimonas* and *Bacillus* [201], *E. coli* [202], and *Lactobacillus bulgaricus* [203] are among the bacteria that have been utilized to synthesis silver NPs.

Investigations into the mechanisms behind the synthesis of silver NPs by bacteria are still necessary. In summary, the synthesis of silver NPs via bacterial-assisted means is an easy, efficient, and sustainable process.

**Table 6:** Classification and comparison of various synthesis methodologies of silver NPs.

Types of Methods	Features	Limitations	Studies
Ball milling [85]	-Cost effective -Utilize at ambient temperature	-Energy-intensive process -Potential of agglomeration -Challenging for uniform NP size distribution -Less suitable for large-scale production	-The size of the silver NPs, according to the particle sizing system measurement results, is roughly 100 nm, which is consistent with the findings from the transmission electron microscopy (TEM) and scanning electron microscopy (SEM) [204]. -According to the experimental findings, silver NPs with a limited size distribution (4–8 nm) can be produced [205].
Laser ablation method [206]	-High purity -Small and uniform NP morphology -Low agglomeration rate	-Energy-intensive process -Low production yield -Complex setup and maintenance -Less suitable for large-scale production -Complex equipment and setup	-A shorter average particle size is found by TEM investigation when the laser is used at a high power of 570 mW for 40 min and a short laser wavelength of 532 nm [207]. -Without the requirement for reducing and stabilizing agents in pure acetonitrile and N,N-dimethylformamide, stable colloidal solutions of free silver NPs (4–10 nm) are produced by laser ablation of the bulk metal [208].
Vapor condensation method [128]	-Appropriate for long-term experiment conditions -Suitable for large-scale synthesis	-Low production yield -Energy-intensive process -Low production yield -Less suitable for large-scale production -Complex equipment and setup	-Helium is flowing within the process chamber when silver NPs are generated utilizing an inert gas condensation technique. Depending on the growth circumstances, the particle size varies between 9 and 32 nm. Particles with a spherical shape and less agglomeration form at lower evaporation temperatures and inert gas pressures are produced [209].
Electrical Arc discharge [142,143]	-High purity -Simple equipment -Simple processing -High synthesis rate	-Simple equipment -Requires significant electrical energy to maintain the arc	-Two identical metallic electrodes placed one millimeter apart in a 100-mL liquid produce the plasma arc-discharge. For silver NPs, the size distributions computed from TEM images show mean particle sizes of 73 nm [210]. -Innovative and simple technique for creating silver NPs (20–30 nm) with a predetermined nanosize and spherical shape that makes use of the arc-discharge method [131].
Chemical reduction [211]	-Simple -Cost effective -Good production rate	-Toxic and hazardous chemicals	By reducing AgNO_3_ with a combination of two chemical agents—sodium citrate and tannic acid—monodisperse silver NPs are produced. Tannic acid and sodium citrate are combined to produce NPs that are uniform in size (approximately 30 nm) and shape [212]. As a reducing agent, 1% trisodium citrate is used for the production of silver NPs. Without utilizing any outside stabilizers or surfactants, the silver NPs with a size of around 103 nm and good dispersion are produced [213].
Electrochemical synthetic method [214]	-Simple reaction control -Less pollutant -Moderate synthesis conditions	-Less suitable for large-scale production	-Two to seven nm silver NPs are produced via an electrochemical process that involves dissolving a metallic anode in an aprotic liquid [154]. -A technique known as electrochemical oxidation/complexation, which is followed by UV irradiation reduction, is used to create distributed chitosan-silver NP. The development of surface plasmon absorbance at about 420 nm indicates the formation of the NPs [215].
Microwave-assisted synthesis [216]	-Time-saving -Efficacy of energy conversion at a high level	-Complex and expensive equipment -Less suitable for scale-up	-Using a microwave combustion process, silver doped lanthanum chromites are synthesized. Through TEM, nanosized particles as tiny as ~7–8 nm and bigger ones ~20–26 nm are seen [217].
Photoinduced reduction [159]	-Utilize at ambient temperature -Safe Chemicals	-Time consuming -Expensive equipment	-Silver nanoprisms (40–220) are produced utilizing a photoinduced process with just three chemical ingredients. The ideal conditions for colloids stability are found using Zeta Potential measurements [218]. -Using several proteins as templates, silver NPs (approximately 8.6 nm) with unique LSPR absorption spectra can be produced upon light irradiation [219].
Microemulsion technique [220]	-Low input of mechanical force	-Susceptible to change -Extensive formulation effort -Low yield	-Using the recovered biosurfactant, silver NPs are synthesized by the microemulsion process, and their properties are assessed through UV-vis spectroscopy, powder-XRD, TEM, and zeta potential. The characteristic UV-vis absorption peak at 440 nm is present in the generated silver NP. The average particle size of the NP is found to be 17.89 ± 8.74 nm using Powder-XRD and TEM investigation, along with its cubic structure [221].
Irradiation method [128]	-Maintenance of synthesis conditions -High purity -Uniform size distribution -Suitable for large-scale production	-Limited reaction flexibility -Potential of agglomeration -Radiation concerns	-Silver NPs, measuring 21.3 ± 7.3 nm on average, are produced by synthesizing 10 mg of chloramine T. Chloramine T concentrations below produce smaller, less stable NPs. The addition of PVP facilitates the formation of larger NPs with diverse shapes, including rods, spheres, and cubes [222]. -The γ-irradiation method creates silver NPs inside the montmorillonite (MMT) interlamellar space without the need for a reducing agent or heat treatment. TEM and X-ray diffraction investigations reveal the creation of face-centered cubic silver NPs with a mean diameter of roughly 21.57–30.63 nm [223].
Plants [136,224,225]	-Simple processing -Wide-ranging applications -Use of safe and non-toxic reagents	-Unknown mechanisms that affect synthesis	-The resulting silver NPs containing *L. acapulcensis* are spherical or quasi-spherical in shape, with an average diameter of 5 nm. Their diameters vary from 1.2 to 62 nm [226]. -Silver NPs are synthesized from the fruit bodies of the plant *Tribulus terrestris* L. Upon observation, it is discovered that the spherical-shaped silver NPs range in size from 16 to 28 nm [227].
Algae [178,179]	-Simple processing -Cost effective -Small and uniform NPs morphology -Use of safe and non-toxic reagents -Eco-friendliness	-Slow synthesis rate -Unknown mechanisms that affect synthesis	-*Caulerpa racemosa*, a marine algae, is used for producing silver NPs. A TEM image reveals the development of silver NPs that range in size from 5 to 25 nm [228]. -Silver NPs mediated by *Sargassum coreanum* (marine algae) are successfully produced. The interlayer distance (d-spacing value) of about 0.24 nm is found, and the generated silver NPs’ deformed spherical form and mean particle size of 19 nm are validated by the high-resolution transmission electron microscopy (HRTEM) pictures [229].
Fungi [192,230]	-Eco-friendliness -Simple processing -Less non-pathogenic behavior -High intracellular uptake	-Unknown mechanisms that affect synthesis -Pathogenic behavior -Process longevity	-A very stable silver hydrosol is produced when aqueous silver ions are exposed to the fungus *Fusarium oxysporum*, causing the ions to disappear in solution. Proteins released by the fungus stabilize the silver NPs, which have a diameter of 5 to 15 nm, in solution [191]. -*Duddingtonia flagrans* (AC001), a nematophagous fungus, is used to produce extremely stable silver NPs. TEM and dynamic light scattering reveal roughly 11, 38 nm monodisperse and quasispherical silver NPs [231]
Bacteria [198,224]	-Simple processing -Eco-friendliness	-Unknown mechanisms that affect synthesis -Slow synthesis rate -Pathogenic behavior -Large size distribution	-The results indicate that *Lactobacillus bulgaricus* has a great deal of potential for producing silver NPs with a size range of 30.65–100 nm [203]. -*Rhodococcus*, *Brevundimonas*, and *Bacillus*—recently identified from a consortium associated with the Antarctic marine ciliate *Euplotes focardii*—are used as reducing and capping agents in the production of silver NPs. Despite not being in contact with one another, the NPs are grouped together and have a spherical to rod-shaped shape. Their diameters range from 20 to 50 nm [201].

### 3.4. Factors Affecting Silver NP Synthesis and Their Stability

#### 3.4.1. Factors Affecting Silver NP Synthesis

**Figure 6:**
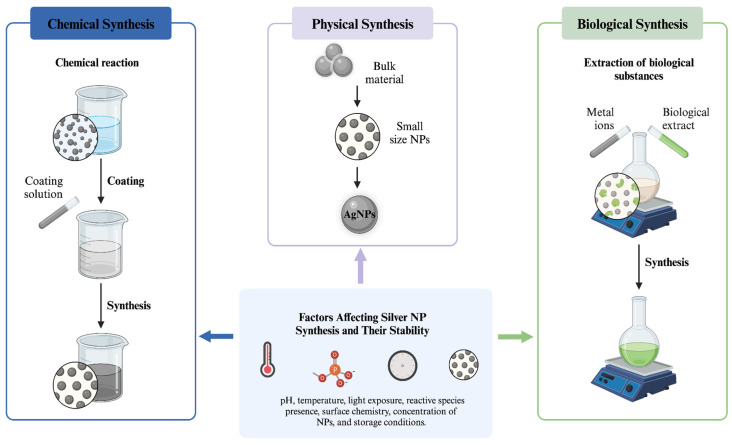
The stability and synthesis of silver NPs—various factors [85,128].

The concentration of the precursor, the reducing and stabilizing agents, the pH of the reaction media, the temperature, the length of the reaction, and the agitation all affect the synthesis of silver NPs (Figure 6). Among these, the concentration of the silver salt precursor plays a crucial role in determining the size and dispersion of the NPs. Higher concentrations of the precursor typically lead to the formation of larger particles. The average crystallite size of silver NPs increased from 23.87 nm to 24.51 nm and ultimately to 25.16 nm in the study of Htwe et al., for example, when the concentration of AgNO_3_ was increased from 0.5 mM to 0.7 mM and eventually to 0.9 mM. This indicates that the initial concentration of metal ions has an effect on the average size of the generated NPs, with larger NPs being created at greater concentrations [232].

The kind and concentration of reducing agents utilized have a big impact on how quickly silver ions are reduced to silver NPs. Ascorbic acid, NaBH_4_, and citrate are examples of strong reducing agents that rapidly produce smaller particles, while softer reducing agents produce larger particles over a longer period of time. In addition, the stability, size, and shape of the particles are greatly influenced by stabilizing chemicals such as citrate, PVP, or PEG. They help prevent agglomeration by maintaining particle dispersion and preventing further aggregation [128]. Variations in particle size and shape can arise from changes in the charge on NPs and the reduction rate due to pH fluctuations in the reaction media. Ondari Nyakundi et al. demonstrated that silver NP synthesis is most effective at pH 7, with optimal particle production occurring at a pH of 7.6. At this pH, the interaction between silver ions and biomass is primarily ionic, as silver acts as an anion in aqueous solutions. Because the biomass has more positively charged functional groups, low pH levels may make it easier for silver ions to approach binding sites.

Silver, a soft metal, binds biomass mostly through amino and sulfhydryl groups, which are thought of as soft ligands and have a stronger positive charge at low pH levels. These groups bind Ag^+^ to Ag^0^. Furthermore, even though carboxylic groups are thought to be challenging ligands, they are protonated at a low pH and may aid in the binding of silver ions. These groups are common in biomass [233].

The kinetics of the reaction are significantly influenced by temperature. Elevated temperatures during synthesis accelerate the reaction rate dramatically, leading to the growth of larger particles and potentially initiating unwanted side reactions. For example, at 50 °C, the reaction mixture rapidly turned brown, resulting in a black powder as the final product. Large, apparently agglomerated particles that depart from a spherical shape were found in the produced silver NPs, according to TEM examination. Furthermore, the highest surface plasmon absorption band sharpened from 90 °C to 110–120 °C, indicating a greater concentration of produced silver NPs. But when the reaction was carried out at 10 °C, it happened very slowly, and the solution’s color didn’t change for three hours [25,234]. Even though numerous studies have shown that synthesis proceeds more quickly at higher temperatures, NP quality must be taken into account [128].

Along with other conditions during NP synthesis, longer reaction durations can lead to the formation of larger particles. This occurs because prolonged reactions result in continued silver ion reduction and deposition on preexisting nuclei, thereby influencing the growth and size of the NPs [233]. Moreover, the NPs’ homogeneity and size distribution are affected by the kind and degree of agitation, including ultrasonication and stirring [128].

#### 3.4.2. Factors Affecting Silver NP Stability

Silver NP stability is influenced by several factors, including pH, temperature, light exposure, the presence of reactive species, surface chemistry, NP concentration, and storage conditions (Figure 6). As mentioned earlier, stabilizers, also known as capping agents, play a crucial role in preventing additional silver NPs from binding to or being absorbed on NP surfaces, which can lead to agglomeration. The effectiveness of these stabilizers in preventing agglomeration and oxidation is highly dependent on their type and quantity. Aggregation can occur due to the screening of electrostatic repulsions between NPs by ionic strength or the presence of salts in the solution. To enhance the stability of silver NPs, researchers have functionalized them with a range of substances, including PEG, chitosan, PVP, PVA, and polystyrene, as well as core-shell structures with gold and alloys with chromium, among other polymers [235,236].

Zeta potential is a commonly used expression to describe the stability of NPs. Because of charge equilibrium, retention with zeta potentials less than 20 mV are considered unstable and have a low ability to aggregate NP [237]. When the zeta potential is between 40 and 60 mV, it is regarded as stable and is measurable in both positive and negative modes. In addition, factors including pH, ionic strength, polydispersity, medium concentration, and zeta potential—which is a measure of dispersion stability—have an impact on the stability of nano-systems containing metallic NPs. It is commonly accepted that smaller, spherical particles and nano-preparations are more stable [238].

While higher temperatures increase kinetic energy and may lead to agglomeration, changes in pH can also affect NP stability by altering its surface charge and zeta potential. For instance, proteins used in NP encapsulation may denature at extremely high temperatures, such as 80 to 100 °C. This denaturation affects Ag+ ion nucleation, causing NPs to aggregate. Additionally, inappropriate conditions reduce enzyme activity related to synthesis, leading to larger NP sizes and decreased stability [190].

Stability is also influenced by the concentration of NPs in a solution, with higher concentrations increasing the likelihood of particle interactions and aggregation. To maintain stability, silver NPs should be stored under optimal conditions, including a constant temperature, protection from light, and an inert environment [128].

## 4. Toxicity

**Figure 7:**
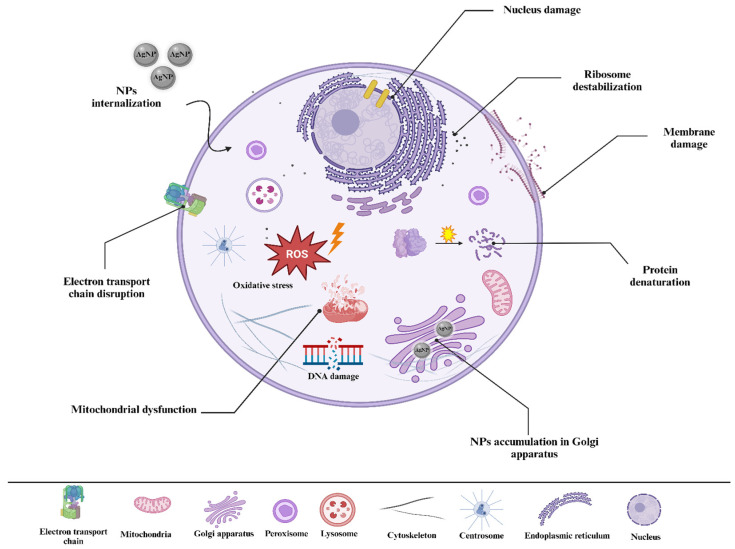
Representation of the toxicity mechanisms of silver NPs. Silver NPs induce toxicity by multiple mechanisms, including internalization, electron transport chain disruption, ROS generation, mitochondrial dysfunction, DNA and nucleus damage, ribosome destabilization, membrane damage, protein denaturation, and accumulation in the Golgi apparatus [241].

Despite their widespread applications, studies in current literature underline the negative effects of silver NPs on living organisms. Their small size allows silver NPs to pass through biological membranes and penetrate cells, subsequently leading to toxicity [239]. Although the exact mechanism remains unknown, it is primarily attributed to the generation of ROS, cellular dysfunction, and inflammation in various tissues. It also disrupts cellular functions and results in adverse health effects, including DNA damage, organ dysfunction, and the development of chronic conditions (Figure 7) [240].

Lee et al. showed that silver NPs increased the production of ROS in NIH 3T3 cells and led to oxidative damage and reduced levels of glutathione. Also, this oxidative stress was found to trigger both autophagy and apoptosis, with silver NPs promoting the formation of autophagosomes and activating apoptotic pathways [242]. Similarly, Choi et al. examined the hepatotoxic effects of silver NPs on adult zebrafish, focusing on the induction of oxidative stress and apoptosis. Findings revealed that exposure to silver NPs caused cellular impairment of liver tissues, supported by histopathological changes and increased levels of malondialdehyde, and increased the expression of pro-apoptotic genes along with signs of DNA damage [243].

Mitochondrial dysfunction is one of the key mechanisms, where the accumulation of silver NPs disrupt ATP production and trigger cell death pathways [244]. Research in this aspect revealed the impact of silver NPs on rat liver mitochondria, demonstrating notable mitochondrial dysfunction. In particular, exposure to silver NPs caused a reduction in ATP synthesis, disrupted mitochondrial membrane potential, and decreased the respiratory control ratio [245]. In addition, Pereira et al. revealed that silver and TiO_2_ NPs, either solely or combined, considerably impacted mitochondrial function in Wistar rats. Their study revealed exposure to these NPs resulted in mitochondrial swelling, along with a decrease in the respiratory control ratio, indicating an impairment of oxidative phosphorylation [246].

Moreover, the toxicity of silver NPs is influenced by variables such as concentration, shape, surface modification, exposure time, and synthesis methods [77]. Perde-Schrepler et al. investigated the size-dependent cytotoxicity and genotoxicity of silver NPs in cochlear cells (HEI-OC1) and keratinocytes (HaCaT). They compared the negative effects of silver NPs with different sizes (5, 25, 50, and 100 nm), on cellular viability, ROS production, and DNA damage. Findings indicated that smaller silver NPs, those with sizes of 5 and 25 nm, exhibited higher toxicity compared to their larger counterparts [247]. Similarly, Park et al. examined the toxicological effects of silver NPs of varying sizes, following oral administration in mice. Over 14 and 28 days, mice were treated with silver NPs of different sizes (22, 42, 71, and 323 nm) to evaluate tissue distribution, serum cytokine levels, and immune responses. Findings indicated that smaller silver NPs were more readily absorbed and accumulated in organs, thereby leading to notable increases in pro-inflammatory cytokines and changes in lymphocyte distribution. Also, it was emphasized that smaller silver NPs (especially measuring 22 nm), were able to penetrate the blood-brain barrier (BBB) more efficiently than larger ones, which may raise concerns about potential neurotoxic effects [248].

Another important factor that influences the toxicity capacity of silver NPs is the concentration during the NP treatment. As an example, the toxic effects of silver NPs, including potential DNA damage and induced apoptosis, were studied in an *in vivo* mice model. Three doses (26, 52, and 78 mg/kg) were administered for 72 h. Silver NP-injected mice exhibited increased serum liver injury markers, such as alkaline phosphatase and alanine aminotransferase, indicating hepatic impairment. Additionally, changes in the liver tissue by necrosis and inflammation were observed. At the highest concentration, an increase in the apoptosis rate and DNA strand breaks in lymphocytes were observed [249]. Nevertheless, silver NPs possess a lower toxicity at decreased concentrations, which might lessen the adverse effects observed at higher concentrations.

Generally, most *in vitro* studies show that lower concentrations of silver NPs (≤10 µg/mL) do not cause severe damage in biological systems. At concentrations up to 50 µg/mL, toxic effects can reach moderate levels, while concentrations higher than 50 µg/mL are often highly toxic. *In vitro* experiments have shown that silver NPs induce high toxicity in HuH-7 cells at 20 and 40 µg/mL, whereas CHANG cells did not show significant toxicity at these concentrations [250]. In addition, when the concentration exceeds 10 µg/mL, severe toxicity is observed, particularly beyond 50 µg/mL in RAW 264.7 cells [251]. Moreover, size-dependent toxicity was demonstrated, with smaller silver NPs (10 nm) exhibiting faster toxicity within the first 24 h compared to larger ones (75 nm). A concentration of 10 µg/mL also induced high toxicity in ex vivo murine precision-cut lung slices and *in vitro* human lung fibroblasts (HFL-1) [252].

To give an example of an *in vivo* perspective of silver NP toxicity, a study demonstrated the effect of silver NPs on fracture healing in a mouse model. The results showed a significant enhancement of osteogenesis by promoting the formation of fracture callus and accelerated gap closure at concentrations of 0.2 mM and 0.4 mM. Conversely, concentrations ranging from 6 μM to 20 μM did not show these results. Moreover, silver NPs at higher concentrations (more than 10 μM) significantly reduced cell viability [253]. Similarly, Ambrožová et al. showed that silver NPs may have varying outcomes depending on their concentration. They found that lower doses of silver NPs (0.25 and 2.5 μg/mL) did not increase ROS levels and activated the Nrf2/HO-1 pathway, thus successfully promoting wound healing. However, higher concentrations (25 μg/mL) demonstrated potential cytotoxicity by increased oxidative stress and decreased nuclear Nrf2 levels [254].

The toxic concentrations of silver NPs differ in environmental studies or when tested on aquatic organisms. The typical tolerable concentration for silver NPs is generally below 10 µg/L. Unlike in general *in vitro* studies (accounting for the change in unit), moderate toxicity levels can still be observed at 100 µg/L. As expected, any concentration above 100 µg/L typically results in high toxicity and a significant reduction in survival rates. A marked reduction in the biomass of the algae Chlorella vulgaris was observed at a concentration of 50 µg/L of silver NPs, with the 40 µg/L concentration showing only a slight difference compared to 50 µg/L [255]. Another study showed that silver NPs had a median lethal dose (LD50) of 62.2 µg/L for *Paratya australiensis* and 62.0 µg/L for Daphnia carinata after 24 h [256]. At 48 h, however, the LD50 for Daphnia carinata was significantly reduced to 35.4 µg/L, while for *Paratya australiensis*, it remained similar at 55.3 µg/L.

Furthermore, silver NPs’ potential toxicity on aquatic environments shows significant potential to harm ecosystem dynamics and diversity [257]. As an example, Liu et al. indicated in their recent review that silver NPs can have ecological risks, particularly for certain organisms such as Daphnia. These organisms play essential roles in freshwater ecosystems. They are also known for being highly sensitive to environmental changes. It was also highlighted that silver NPs can interrupt ecological processes by affecting the survival, growth, and reproduction of Daphnia through oxidative stress induction and genotoxicity [258].

Considering the wide-ranging application of silver NPs, it is important to conduct risk assessments to develop safer NP applications and minimize these potential adverse effects.

## 5. Conclusions and Future Trends

Silver NPs’ increasing popularity emphasizes the importance of understanding their properties and synthesis methods comprehensively. These NPs demonstrate distinct physical and chemical characteristics that are influenced by their size, shape, surface charge, and way of synthesis. Their small size and high surface area to volume cause unique characteristics, including electrical and thermal conductivity, antibacterial activity, and optical features. In addition, these properties enable silver NPs to release silver ions more efficiently, thereby interfering strongly with microbial membranes and providing an enhanced antimicrobial action. Most studies indicate a preference for small-sized silver NPs in these applications for enhanced bactericidal activity and favorable cell viability results. The size of the particles significantly influences the distribution of the NPs in applications. As an example, the small-sized NPs are desirable in bioimaging applications due to the increased cellular accumulation and enhanced LSPR properties. On the other hand, larger-sized NPs that exceed certain threshold values in wound healing studies are more preferable to control the movement of the particles in the tissue. Other common applications of silver NPs are not so different. The high efficiency of small-sized silver NPs in antimicrobial studies has been highlighted multiple times. Size determination takes higher priority if the NP is desired to be used for constructing nanocomposites or for dual-treatment with other types of molecules. Both the aggradation potential and the stability of silver NPs are heavily influenced by their size, whether combined or used alone. Considering the significant potential of silver NPs in nanotechnology and their compatibility with many systems and different nanomolecules, it is clear that the size of silver NPs should be carefully controlled during the synthesis process.

The surface of silver NPs also has a significant role in both their manufacture and uses. The surface charge can influence the molecular interactions between the particles and cells, including bacteria and viruses. Most importantly, silver NPs can be functionalized through surface modification for multiple purposes. This functionality makes them one of the leading particles in drug delivery systems, bioimaging, sensor applications, and more. In drug delivery, surface modifications can improve the bioavailability, safety, and efficiency of molecule transport. Functionalized silver NPs can enhance the specificity of targeting, decrease the possibility of aggregation, and increase the stability of the delivery system through modification with various ligands and biomolecules. The same enhancements are also effective in sensing and imaging applications. Cellular targeting in bioimaging is significantly improved by surface-modified silver NPs. Their characteristics as effective carriers and their unique optical properties are additional advantages for both applications. However, to achieve the desired flexibility in surface modifications, optimizing synthesis procedures remains a top priority

Furthermore, the toxicity of the particles can be affected by charge-induced molecular interactions. However, it can be manipulated to increase the stability and efficiency of the synthesis process by decreasing aggregation of the particle. Surface modification is also essential in bioimaging and biosensing applications. It strengthens cell penetration, specifies cellular targeting, and enhances imaging efficiency. Also, LSPR plays an important role for imaging and sensing applications, while their tunable surface chemistry allows for a targeted functionalization. This versatility optimizes their interactions with biological systems and enhances their efficiency for diagnostics medical and technological applications. To determine the characteristics of silver NPs, the synthesis process needs to be considered. The properties of silver NPs directly influence the performance and applicability of these particles. This is why precisely controlling the synthesis parameters is essential to direct NP application into specific areas. For example, the physicochemical characteristics of silver NPs and their applications are greatly influenced by the chosen synthesis technique. Even though chemical and physical approaches can be utilized, they generally lead to limitations, such as complex purification procedures and excessive energy consumption. However, biological processes, even though they require a more cautious approach and longer processing time, offer more economical and environmental options. The stability, size distribution, and purity of the NPs are further impacted by the choice between top-down and bottom-up methods. The conditions applied during the synthesis of NPs are critical in determining the properties of the particles. Factors such as extract concentration, pH, exposure time, and temperature are essential in NP synthesis. These parameters need to be thoroughly investigated and optimized in existing biosynthesis methods to obtain the desired NP characteristics. In addition, the chemicals and biomolecules used as reducing and capping agents require thorough investigation. These stabilizers are essential for preventing agglomeration during synthesis and must be non-hazardous.

Up to this point, utilization of silver NPs has become one of the main topics in the field of nanomaterials. Considering the comparative studies focusing on the effect of the size and shape of silver NPs in antimicrobial activity, along with eco-friendly approaches such as green-synthesis methods utilizing various plant extracts, comprise one of the biggest portions of the recently created patents. Considering the wide-ranging applications of silver NPs, methods and approaches that reduce toxicity risk are crucial. The current literature presents extensive research, predominantly focused on green-synthesized silver NPs. The preference for green synthesis methods of silver NP production highlights the need for detailed investigation in this area. These methods should be developed for the low-cost, non-toxic, and eco-friendly synthesis of highly biocompatible silver NPs, particularly for applications where toxicity is a critical concern. In this context, the synthesis of silver NPs from plant extracts is one of the most suitable eco-friendly approaches for further research and industrial applications. For instance, the efficiency of plant-based synthesis of NPs, their time-saving procedures, and their capability to create highly bioactive products are currently emphasized in the literature. In recent years, the biosynthesis of silver NPs, especially from plant extracts, has been predominantly used in research. However, the complete optimization of these methods, particularly for industrial and large-scale applications, is still lacking.

The requirements of further research strongly emphasize optimizing the application and applicability of silver NPs, as well as mitigating their adverse effects. From another perspective, since antimicrobial resistance is becoming one of the major health concerns, the search for possible effective alternatives continues. In this manner, silver NPs are thought to replace the traditional antimicrobial agents, as they exhibit potent microbicidal effectiveness. This effectiveness would extend to commercialization processes, such as disinfection solutions or silver NP included-wound dressings, to fight against multi-drug-resistant bacteria.

In addition to these properties, silver NPs’ toxicity should also be evaluated, given the significant number of studies in the current literature. Due to their high antimicrobial activity, silver NPs have the potential to be widely used in various therapeutic applications in the future. Concurrently, their use in food packaging and dental applications, leveraging their antimicrobial properties, further emphasizes the need to clarify their potential for toxicity. Area-specific toxicity standards for silver NPs need to be determined to advance their applications to higher levels. Although some standards exist for testing the toxicity of silver NPs *in vitro* and, to a limited extent, *in vivo*, further developments are necessary to advance these applications in human studies. Since both the properties and synthesis methods of NPs significantly influence toxicity, detailed investigations and standardized methods could accelerate toxicity research for silver NPs.

Considering these, examination and management of their potential adverse effects are necessary to ensure safer outcomes. These attributes collectively highlight the important role of silver NPs in nanotechnology, where their unique combination of properties not only supports innovative uses but also underscores the importance of further research.
